# Next Generation Sequencing Based Forward Genetic Approaches for Identification and Mapping of Causal Mutations in Crop Plants: A Comprehensive Review

**DOI:** 10.3390/plants9101355

**Published:** 2020-10-14

**Authors:** Parmeshwar K. Sahu, Richa Sao, Suvendu Mondal, Gautam Vishwakarma, Sudhir Kumar Gupta, Vinay Kumar, Sudhir Singh, Deepak Sharma, Bikram K. Das

**Affiliations:** 1 Department of Genetics and Plant Breeding, Indira Gandhi Krishi Vishwavidyalaya, Raipur 492012, Chhattisgarh, India; parmeshwarsahu1210@gmail.com (P.K.S.); richasao.agro@gmail.com (R.S.); 2 Nuclear Agriculture and Biotechnology Division, Bhabha Atomic Research Centre, Mumbai 400085, India; suvendu@barc.gov.in (S.M.); gtmvish@barc.gov.in (G.V.); skgupta@barc.gov.in (S.K.G.); sudhirs@barc.gov.in (S.S.); 3 Homi Bhabha National Institute, Training School Complex, Anushaktinagar, Mumbai 400094, India; 4 ICAR-National Institute of Biotic Stress Management, Baronda, Raipur 493225, Chhattisgarh, India; vinay.nibsm.cg@gov.in

**Keywords:** NGS, induced mutagenesis, point mutations, forward genetics, bioinformatics, mapping, causal mutations

## Abstract

The recent advancements in forward genetics have expanded the applications of mutation techniques in advanced genetics and genomics, ahead of direct use in breeding programs. The advent of next-generation sequencing (NGS) has enabled easy identification and mapping of causal mutations within a short period and at relatively low cost. Identifying the genetic mutations and genes that underlie phenotypic changes is essential for understanding a wide variety of biological functions. To accelerate the mutation mapping for crop improvement, several high-throughput and novel NGS based forward genetic approaches have been developed and applied in various crops. These techniques are highly efficient in crop plants, as it is relatively easy to grow and screen thousands of individuals. These approaches have improved the resolution in quantitative trait loci (QTL) position/point mutations and assisted in determining the functional causative variations in genes. To be successful in the interpretation of NGS data, bioinformatics computational methods are critical elements in delivering accurate assembly, alignment, and variant detection. Numerous bioinformatics tools/pipelines have been developed for such analysis. This article intends to review the recent advances in NGS based forward genetic approaches to identify and map the causal mutations in the crop genomes. The article also highlights the available bioinformatics tools/pipelines for reducing the complexity of NGS data and delivering the concluding outcomes.

## 1. Introduction

Availability of abundant genetic variability and diversity in the gene pool is the elementary need for genetic enhancement of any crop species. Conventional plant breeding is entirely dependent on the accessibility of sufficient genetic variations for crop improvement. However, the required genetic variations may not always be available in proper form [1,2]. Crop improvement through conventional plant breeding might be hampered due to a complicated and long breeding cycle, availability of the narrow gene pool, reduced vigor, and fertility [3]. In such situations, induced mutagenesis may play a greater role in crop improvement as it may create huge genetic variations and new alleles for economically important traits within a short period than the conventional one [4]. Induced mutagenesis rapidly increases the frequency of genomic variation with minimal reduction in viability as compared to spontaneous mutations. The induced mutant alleles may serve as the source of genetic variation for crop improvement and candidate gene discovery through functional genomics in many crops [5]. Varieties developed through induced mutagenesis are similar to immediate parent except for one or few traits and have proven to be environmentally acceptable, safe, and non-hazardous. Nowadays, mutation techniques are frequently used by plant breeders to discover new genetic sources for biotic and abiotic stress tolerance, herbicide resistance, nutritional quality traits, yield potential, and climate resilience [1].

Induced mutagenesis has also been proven to be an efficient tool for the discovery and mapping of important genes/quantitative trait loci (QTLs), exploring gene function and for dissection of molecular, biochemical, and metabolic pathways involved in the development of mutant phenotype [6]. Basic understanding about genetics, regulatory mechanisms, and functional genomics of the mutated genemay potentiate the avenues for genetic improvement of crops through new breeding techniques [3,5]. Determination of gene function by associating candidate genes with their phenotypes is commonly known as forward genetics. Forward genetic screening requires only a desirable phenotype to explore the causal mutations/genes/QTLs through genetic mapping, fine mapping, positional cloning, and other advanced genomic approaches [7,8]. In order to identify the causal mutations, positional cloning was conventionally used which is a tedious, costly, and time-consuming process due to low recombination rates in the target regions [9]. The recent advancements in forward and reverse genetic technologies have expanded the applications of mutation techniques in advanced genetics and genomics, ahead of direct use in breeding programs [7]. The advent of next-generation sequencing (NGS) has enabled the identification of causal mutations in a short period of time and at relatively low cost [10]. Availability of sequence variants in a sample of interest can be determined by comparing their genome with an available reference genome or without comparing with reference genome, by the help of bioinformatics approaches [8]. In this way, NGS based forward genetic screen is very useful for the identification and mapping of causal mutations.

To accelerate the mutation mapping for crop improvement, several high-throughput and novel NGS based forward genetic approaches such as SHOREmap [11]; Next-Generation Mapping (NGM) [12]; MutMap [13]; deep Candidate Re-sequencing (dCARE) [14]; MutMap+ [15]; MutMap-Gap [16]; RNA Sequencing based mapping [17]; QTL seq [18]; Exome Capture [19,20]; Needle in the k-stack (NIKS) [21]; MutChromSeq [22]; MutRenSeq [23]; Simultaneous Identification of Multiple Causal Mutations (SIMM) [24]; Targeted Chromosome-based Cloning via Long-range Assembly (TACCA) [25]; AgRenSeq approach [26]; Longer needle in a scanter k-stack (LNISKS) [27] and many more have been developed and validated by researchers in the past 12 years. These techniques are cost effective and highly capable to identify causal mutations/desired genes and gene function in any crop mutant within a short period of time. However, data obtained from the NGS of any crop genome are highly complexed which requires more sophisticated data processing [7,28]. NGS based forward genetic approaches are vastly reliant on compatible bioinformatics tools and pipelines to obtain the final outputs and desired information. Numerous bioinformatics tools and pipelines such as MAQGene [29]; GenomeMapper [30]; Mapping and Assembly with Short Sequences (MASS) [31]; Next-Generation Mapping (NGM) [12]; The SNPtrack tool [32]; CloudMap [33]; CandiSNP [34]; SIMPLE Pipeline [35]; artMAP [28] have been developed to perform the different functions. Utilization of these tools/pipelines depends on the NGS methodology, type of experimental materials, and the genome of organisms.

In the post genomic era, induced mutagenesis has been merged with molecular marker, genetic mapping, and sequencing techniques to discover novel candidate genes and validate their specific biological functions with functional genomics. NGS techniques have great potential in metagenomic and agri-genomic research with increased prospects for their utilization in plant breeding. This article intends to review the recent advances in NGS based reference-dependent and reference independent forward genetic approaches to identify and map the causal mutations in the crop genomes. The review article also highlights the available bioinformatics tools and pipelines for reducing the complexity of NGS data and delivering the concluding outcomes.

## 2. Mutation Breeding for Crop Improvement

**Figure 1:**
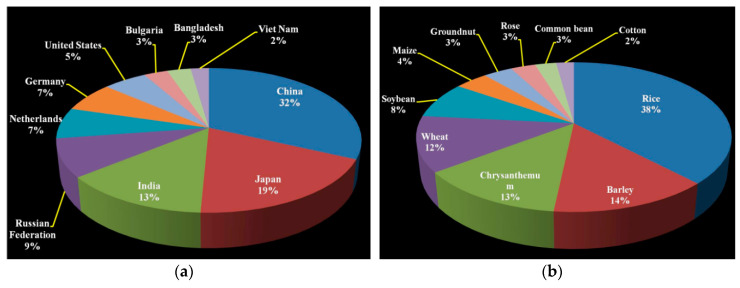
(**a**) Pie chart representing the percentage contribution of top ten countries for crop mutant variety development. (**b**) Pie chart representing the percentage contribution of the top ten officially registered mutant crop species. Common name of the crop is used for grouping purpose. Data came from FAO/IAEA Mutant Variety Database [39].

The discovery of X-rays for increasing the mutation rate in Drosophila by Mullar in 1927 and in barley by Stadler in 1928 has paved the way of modern mutation breeding and genetic studies [4,36]. Mutations are the primary source of all genetic variations existing in all organisms, including plants. The consequential fixed and heritable variations provide the basis for natural selection and evolution [37]. Spontaneous mutations are rare and slow in nature, which made them difficult to exploit in crop improvement. In contrast, mutations induced by physical mutagens such as gamma rays, X-rays, UV light, and charged particle radiation, including fast and thermal neutrons, beta and alpha particles, or chemical mutagens such as alkylating agents, intercalating agents, and sodium azide are easy to use and generate about 1000 times more variability than spontaneous mutations [6]. Freisleben and Lein [38] coined the term mutation breeding (Mutationszuchtung) for deliberate induction and development of new mutant lines for crop improvement. Availability of abundant variation for the desired trait is essential for developing a genetically stable and commercial variety through rigorous selections and stabilization process of mutation breeding [37].In the past few decades, several superior crop varieties have been developed through induced mutagenesis by various countries, which made significant impact on food production and consumption [3,36]. By 2020, about 3332 mutant varieties in about 190 crop plants have been developed and uploaded in the FAO/IAEA Mutant Variety Database (http://https://mvd.iaea.org/) [39]. The Asian countries have developed the largest numbers of mutant varieties especially in the small grain cereal crops [39]. China has developed the highest number of crop mutants, followed by Japan and India. About 50% of the total crop mutant varieties were developed by these three countries (Table 1 and Figure 1a). In the world, maximum crop mutants were developed for rice followed by barley and chrysanthemum. More than 70% crop mutants belong to cereal crops (Table 1 and Figure 1b).

Looking to the importance of induced mutagenesis, Indira Gandhi Krishi Vishwavidyalaya (IGKV), Raipur-492012 (C.G.), India and Bhabha Atomic Research Centre (BARC), Mumbai-400085 (M.S.), India is working together for improvement and revival of traditional rice landraces of Chhattisgarh state, India since the year 2012. In the way, more than 300 popular rice landraces has been targeted for their revival and improvement through radiation induced mutation breeding [40,41]. Till now more than 18 stable rice mutants have been developed in the background of 15 popular rice landraces. Several mutant lines are under various stages of stabilization and evaluation. Moreover, three mutant varieties viz., Trombay Chhattisgarh Dubraj Mutant-1 (TCDM-1), Vikram-TCR and Chhattisgarh Jawaphool Trombay has been released. From which, TCDM-1 has been notified by Government of India for commercial cultivation in Chhattisgarh state, India and rest two varieties are under the process of notification [41,42]. Moreover, the mutant TCDM-1 is becoming very popular among the farmers’ of Chhattisgarh state. Dubraj, the parent of TCDM-1 was famous for their aromatic short grains and excellent cooking quality however, it was disappeared from farmers’ field due to tall stature, late maturity and poor yield potential [40,42]. Similarly, Vikram-TCR (parent: Safri-17) is popular for higher yield potential, drought tolerance ability and excellent puffed rice making quality, in addition to semi-dwarf stature and mid-early maturity habit. With the help of mutation breeding, we have reduced the plant height, maturity duration and increased the yield potentials of these mutants [41]. Therefore, mutation breeding is a powerful approach to improve one or two undesirable traits in crop plants [40,41].

A major bottleneck in plant mutation breeding is the essentiality of generating and evaluating large mutant populations to increase the possibility of getting a desirable mutant [6]. This problem may be conquered by site-directed mutagenesis, the process of creating a mutation at a target site in a DNA molecule; and insertion mutagenesis, the insertion of T-DNA or activation of transposable elements, cisgenesis [43,44,45,46]. However, these site-directed approaches require high technicalities and genetic engineering expertise along with the amenability of plant species for tissue culture and callus differentiation [4].

## 3. Need of Identification and Mapping of Causal Mutations

Mutagens create genome wide DNA variations viz., single base substitutions, deletions, inversions, translocations etc. in crop plants. Of the several mutations, a single mutation or a few of them determine the function of the mutated gene. Different approaches through NGS techniques and analysis pipelines help in pinpointing such mutations through the usage of mutant-derived populations. Thus, the idea of applying such NGS based pipelines to correctly identify the causal mutations will help us to understand the nature of mutations, identifying genes, and the impact of such genes on phenotypes.

After establishing the facts on the nature of mutations and mutant genes, some gene specific markers may be generated to transfer the mutant trait in the background of high yielding genotypes with the help of marker (gene-based) assisted breeding (MAB). Such markers for climate resilient mutant traits will be really helpful to transfer the mutant trait even in absence of sophisticated screening facility in field. Transfers of *sub1* and salt tolerant gene in rice are the prominent example of such efforts [4,47]. Moreover, understanding of casual mutations will also assist us to apply the same knowledge for improvement of traits of a mega-variety through the use of new breeding technologies within a short span of time.

## 4. Concept of Mapping, Sequencing, Resequencing, and Mapping by Sequencing

Genome mapping is an important tool for sequential allocation of loci/genes along the chromosomes and to determine the relative distances between them. Genetic linkage mapping and physical mapping are two forward genetic approaches for genome mapping. Genetic linkage mapping method involves constructing genetic maps to show the relative position of genes/polymorphic markers along the chromosomes and is based on the Mendelian principles of segregation and recombination. The first genetic linkage map was constructed in 1913 for fruit fly (*Drosophila melanogaster*) using phenotypic markers [48]. Since then, morphological markers have been used for genetic mapping in many studies. However, morphological markers have limited polymorphism and are strongly influenced by environment, making them less useful in plant breeding. With the advent of DNA technology, different kinds of molecular markers such as restriction fragment length polymorphism (RFLP), random amplified polymorphic DNA (RAPD), Inter simple sequence repeat (ISSR), microsatellite or simple sequence repeat (SSR), amplified fragment length polymorphism (AFLP), single nucleotide polymorphism (SNP) have been identified and deployed to accelerate genetic mapping studies in crop plants [49].

Most of the agriculturally important traits in crop plants are complex or quantitative in nature. Accurate and well-saturated genetic maps may serve as an important tool in genetic and genomic analysis of complex traits [50,51]. Furthermore, genetic mapping is a pre-requisite to facilitate high-resolution genetic mapping, map-based cloning, and construction of physical maps. Multiple genetic maps are currently available for most of the important crop species including cereals [52,53,54], legumes [55,56,57], oil seeds [58,59,60], etc. Genetic mapping requires the generation of large segregating populations by crossing two parents having contrasting phenotypic differences for one or more traits of interest. Different types of segregating populations that can be utilized for genetic mapping usually consist of F_2_ populations, backcross (BC) populations, recombinant inbred lines (RILs), and double haploids (DH) [50].

For genetic linkage mapping, Michelmore et al. [61] developed a simple and rapid method called bulked segregant analysis (BSA) to identify the molecular markers linked to the gene of interest. In BSA, two DNA bulks contrasting for the target trait are prepared from a segregating bi-parental population and screened with molecular markers to identify the polymorphic markers that distinguish the two bulks. Based on segregation analysis, the identified polymorphic markers are then mapped to the target gene to identify the precise genetic distance between the markers and the gene. These genetic distances are calculated based on recombination frequency between the markers and gene, and are usually expressed in Centimorgan (cM). BSA is the most widely used method to map the genes controlling simple traits in plants, and can also be applied to genetic dissection of the QTLs by screening bulks of informative individuals. BSA approach has been successfully employed to map many agronomically important traits in crop plants [62,63,64,65,66,67].

While genetic mapping provides the location of the target genes/loci, physical mapping is required to get an estimation of the actual (physical) distance between loci/genes on a chromosome. A physical map consists of linearly ordered array of genomic DNA fragments encompassing the whole genome or a particular genomic region of interest. The physical distances between loci are expressed as the number of base pairs between them. For physical mapping, large-insert genomic DNA libraries constructed using high capacity vectors such as yeast artificial chromosome (YAC), bacterial artificial chromosome (BAC) and cosmids are required and have been constructed for most of the major crop and model plants [68,69,70,71,72,73,74]. Physical maps are important genomic resources for map based gene cloning, analyzing chromosome and genome structure, and establishing relationship between genetic (cM) and physical distances (bp).

DNA sequencing has been a high priority in genetics research to determine the sequence of individual genes, larger genomic regions, full chromosomes, or entire genomes of any organism. Initially, Sanger sequencing (chain termination method) and Maxam-Gilbert sequencing (chemical degradation method) were the two methods developed for DNA sequencing [75,76]. Because of its high efficiency and certain other advantages, Sanger sequencing gained importance and became the most preferred and widely used DNA sequencing technique among the biologists. In this method, read length up to 1000 bp can be obtained with an accuracy of 99.99%. With the development of automated Sanger sequencing platforms, Sanger sequencing has been extensively used for construction of reference genomes of several plant species like Arabidopsis [77], rice [78], maize [79], sorghum [80], and soybean [81]. However, high operational costs, low throughput, and longer time to output have limited the application of Sanger sequencing for whole genome sequencing (WGS) in many species, particularly in those having large genome. The emergence of next-generation sequencing (NGS) technologies has reduced the sequencing cost several fold and greatly increased sequencing throughputs. Over the past 12 years, advances in NGS technologies haveled to the development of many commercial NGS platform such as Roche/454 (GS20, GS FLX, GS FLX Titanium, GS Junior and GS Junior+), Illumina/Solexa (MiniSeq, MiSeq, HiSeq and HiSeqX), ABI/SOLiD (5500 W, 5500×lW), Ion Torrent (Ion PGM, Ion Proton, Ion S5), PacBio and Oxford Nanopore (MinION) [82,83]. NGS technologies utilize massively parallel sequencing from multiple samples at much reduced cost to generate several giga bases of sequence information per day with minimum error rate [84].

In species where WGS are available, NGS technologies allow re-sequencing of the genome rapidly by using already available genome sequence as a reference to guide the alignment of the reads. The re-sequencing facilitates identification of minor sequence variations such as SNPs and insertions/deletions (InDels) between the reference genomes and the sample of interest by comparing the consensus sequence, leading to rapid mapping and identification of desirable mutations [85,86].

## 5. Role of NGS in Detection and Mapping of Mutated Genes/Locus

Mutagenesis-based screens are a powerful tool to identify novel genes or gene functions. Though it is not difficult to generate mutants using various chemical or physical mutagens, the molecular identification and characterization of genes involved in the altered phenotype/biological processes remain the primary goal of such mutant analyses. Identification and mapping of desired mutations in any genome involve several steps viz., genetic mapping of a wide chromosomal region having gene of interest (mutated genes); (i) identification of candidate genes (mutated genes) contained in the identified chromosomal region; and (iii) validation of identified candidate genes responsible for the mutant traits.

In the past, various strategies including transposon- or transgene-tagged mutagenesis, molecular markers were tried to find out the mutated gene [87]. But, as mutation happens randomly, conventional DNA markers can define the wide or large chromosomal region having the desired mutation. Generation, identification, and characterization of mutants have always been an important part of plant breeding. However, identification of the causal gene for the mutant phenotype by classical linkage analysis and map based cloning is a costly, laborious, time-intensive task and imposed a significant limitation. Therefore, targeted sequencing within the selected chromosomal region may be required to identify the true causal mutation. The limitations of positional cloning and linkage mapping have been resolved with the advent of next generation sequencing. It may serve as an important tool for easy and rapid identification and mapping of the causal mutations through whole genome sequencing [88,89]. It has greatly improved the power and efficiency of mutant identification that not only allows the identification of genetic markers but also enables for the simultaneous identification and mapping of causal mutations. In the last decade, the method of mapping and cloning the mutations of interest has advanced quickly with the advent of NGS-based approaches [8,11,88,90].

NSG technologies have already gained widespread popularity in plant breeding. Apart from whole genome sequencing, NGS along with powerful computational pipelines have provided novel and rapid ways for transcriptome sequencing, molecular markers discovery, gene expression studies, and targeted re-sequencing to identify agronomically important genes in plants [91,92,93,94]. The NGS helped to sequence whole genome in a matter of weeks rather than years and in turn, led to the development of several NGS-based mapping approaches in plants that have significantly reduced the time required for mutation identification [11,12,95]. The approaches that primarily rely on the principle of ‘mapping by sequencing’ have reduced the efforts and time required to identify the causal mutations [11]. Many mapping by sequencing approaches that combines classical mapping strategies with NGS have been successfully applied to directly map causative mutations in plants [11,12,13,14,15,16,17,18,19,20].

## 6. NGS Based Forward Genetics for Identification and Mapping of Causal Mutations

Once a mutant is generated, it is important to know which genes have been altered to induce the phenotype of interest. In this context quantitative trait locus (QTLs) and map-based cloning have been proved as effective forward genetic approaches to characterize point mutations or small insertions/deletions (InDels). Once a link between QTL and the quantitative trait is established, it may be further mapped or cloned individually to identify the gene of interest. Depending upon the size of QTL, fine mapping, association mapping, or positional cloning are further performed with large number of molecular markers to identify the candidate gene responsible for the trait of interest [96]. The NGS based forward genetic approaches have followed the three main approacheswhich are briefly described below:

*(a) Genotyping by sequencing (GBS) approach*

Among various NGS platforms, genotyping by sequencing (GBS) has significantly increased the availability and applicability of molecular markers for crop improvement. GBS helps in the identification and genotyping of a huge number of SNPs. Candidate SNPs may be linked with desired traits with the help of genome-wide association mapping and/or QTL mapping and further utilized in marker-assisted breeding for gene introgression or to validate trait-linked haplotypes in crop plants. GBS approaches were used for identification and mapping of genes/QTLs in recombinant inbred lines of rice [97,98], maize, and barley [94] and double haploid population in wheat [99]. Though GBS does not necessarily require a reference genome, the two major drawbacks viz., intrinsic error rate of sequencing process and low depth of sequencing areassociated with this approach [100].

*(b) Whole genome resequencing (WGR) approach*

With the availability of whole genome sequence as a reference for many commercial crops, the whole genome resequencing (WGR) has gained a lot of importance. In this approach, sequencing of a new individual is performed and compared with its reference genome to identify not only polymorphism (including SNPs) but also structural variants like insertions-deletions (InDels), gene conversions, etc. [92]. Using this approach, constitutive photomorphogenic-9 (*COP 9*) signalosome complex subunit 8 (*CSN8*) genes responsible for seed weight in chickpea wereidentified. Similarly, the WGR was effectively utilized for identification of the desired gene in other crops including *Setariaitalica* [81] and rice [86].

*(c) RNA-Seq approach (whole exome sequencing approach)*

RNA-Seq i.e., direct sequencing of cDNA derived from total transcripts has turned out to be an important tool for comprehensive profiling of QTL genes expression. This approach focuses on the protein coding regions in the genome, comprising approximately 1–2% of the genome [101]. NGS assisted expression profiling in a mutant and its comparison with the parent can identify candidate genes associated with the desired phenotype. In sorghum (*Sorghum bicolor* L.), comparative RNA-Seq analysis of parents revealed 108 differentially expressed genes (DEGs) were involved in plant hormone metabolism, glycolysis and nitrogen metabolism [102]. These DEGs were situated near to QTLs of multiple agronomic traits under normal and low-nitrogen conditions. Similarly, RNA-Seq analysis and QTL-analysis jointly helped to identify the gene of interest in maize [103] and *Glycine max* L. [104]. Further, integration of both the approaches i.e., RNA-Seq and QTLs (called expression QTLs or eQTLs) enabled the expression of complex traits [105,106].

By following these methodologies, numerous NGS based forward genetic approaches have been developed and applied in various crops for identification and mapping of causal mutations (Table 2). Most of the approaches followed the principles of mapping by sequencing of bulk segregants of two populations. However, minor differences in type of base materials to be used, need of reference genomes, the imposition of custom filters, utilization of bioinformatics tools/pipelines, etc. have been found in all the NGS based approaches (Table 3). The brief description of these NGS based forward genetic approaches will be discussed individually in the subsequent paragraphs.

### 6.1. SHOREmap

SHOREmap was developed by Schneeberger et al. [11] to identify the causal mutations. It helps in genome wide genotyping and sequencing of a candidate gene from a large pool of recombinant lines.

It follows the principle of mapping-by-sequencing. Selfing of mutagen induced M_1_ lines yields M_2_ population from which desired mutant is selected. This recessive mutant (after confirming its true breeding behavior) is crossed with genetically diverse wild-type line followed by selfing to produce F_2_ progenies. Resultant F_2_ population will segregate for the mutant phenotype. The individuals displaying mutant phenotypes (~500) can be isolated, pooled, and sequenced up to genome coverage of 22×. An ‘interval’ plot is also created. The relative allele frequencies of the two mapping parents can be represented by an ‘interval’ plot which is obtained after analysis revealing the candidate region harboring site of mutation. INTERVAL generates a visual output which allows the user to define a mapping interval. By default, INTERVAL generates 10 different plots of all chromosomes by sliding window analysis. This plot(s) may contain candidate region causing mutation which can be used as an input for ANNOTATE [11,107]. The highest peak must be included within the selected region to select the smallest possible interval. ANNOTATE uses this peak to emphasize the mutations within the interval. A set of software tools have been developed for the analysis of whole-genome sequencing data obtained from SHOREmap process. Currently, SHOREmap v3.0 is useful for analyzing the WGS data [107].

Schneeberger, et al. [11] made a crossing in *Arabidopsis thaliana*, between a slow growth light green leaves mutant in the Columbia-0 (*Col-0*) accession with a wild-type plant in the Landsberg erecta (*Ler-1*) accession and used 500 mutant F_2_ progenies to identify the causal mutation in one next-generation sequencing run of 20× coverage of the genome. Changes in amino acid sequences occurred i.e., Amino acid serine changed into asparagine, due to codon change in the *AT4G35090* gene. However, some limitations are also associated with SHOREmap. Crossing the mutant to highly diverse line create disturbances by altering or interfering with the mutant phenotype, resulting into incorrect phenotyping/pooling, and thus leads to formation of a considerably larger mapping interval. Compared with the other NGS techniques like MutMap, SHOREmap has higher noise in SNP calling and poor alignment. Moreover, F_2_ progenies required for bulking in SHOREmap is much larger than other techniques [142].

### 6.2. NGM (Next-Generation Mapping)

**Figure 2:**
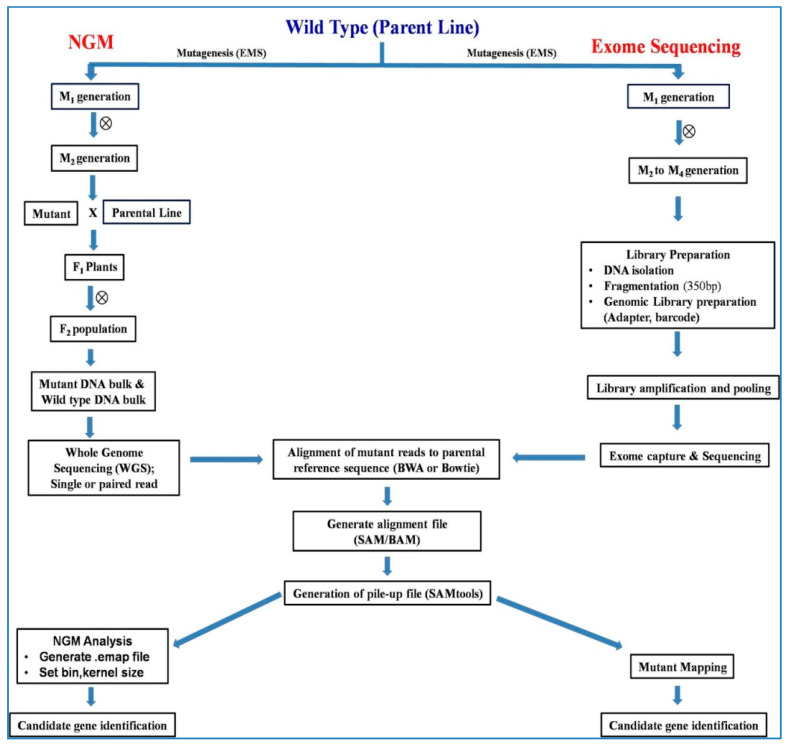
Flow diagram showing the major steps of next generation mapping (NGM) and exome sequencing approaches of forward genetic screen.

NGM is an alternative method to SHOREmap which requires a much smaller mapping population (10–50 F_2_) to identify and map the causal mutations [12]. The NGM approach utilizes the genetic phenomenon that linkage disequilibrium (LD) between a mutant locus and its surrounds genomic region will lead to reduced heterozygosity in that particular region in mapping population like F_2_. Using sequencing and computation tools, a measure of ratio of homozygous and heterozygous locus in the local area is done to identify all type of variations viz., causal mutation, insertion, deletion, integration of T-DNA, etc. The approach was demonstrated to identify three mutant genes in Arabidopsis. The method can identify all type of mutation (insertion, deletion, and SNPs) present in homozygous state causing mutation. Pooled DNA of F_2_ lines depicting contrasting phenotypes were sequenced using illumina paired end sequencing (Figure 2). The resulting sequences were aligned with the Arabidopsis reference genome (TAIR9 release) to identify SNP variation [77]. Localization of identified SNP on genomic regions was carried out to identify hotspot for variation and non-recombinant blocks [12].

A mathematical score termed ‘discordant chastity’ statistic (Ch_d_) derived from the illumina chastity statistic was used to estimate the proportion of reads at these SNP sites which were different from the reference genome. Using the Ch_d_statistic, probability density estimates and linkage analysis, the causal SNP can be pinned down. Compared to map based cloning which requires 100–200 F_2_ population, NGM may be carried out using 10–50 F_2_. Even in cases where reduced representation of genome is available for sequencing (e.g., exome) NGM can be utilized for mutant trait identification. NGM analysis can directly take input from mapping output generated from common packages like Mapping and Assembly with Quality (MAQ) or SAMtools. The approach has been demonstrated to identify causal SNP which are missed by other pipelines and is highly cost and time effective. NGM in combination with map based cloning has been applied in many systems for identification of mutated gene. The NGM approach is flexible and can be utilized for mutant locus identification irrespective of type of mutation viz. SNP, insertion, deletion etc., as LD between mutant locus and surrounding region will lead to reduced heterozygosity regardless of type of mutation [12,143,144].

### 6.3. dCARE (Deep CAndidate RE-Sequencing)

**Figure 3:**
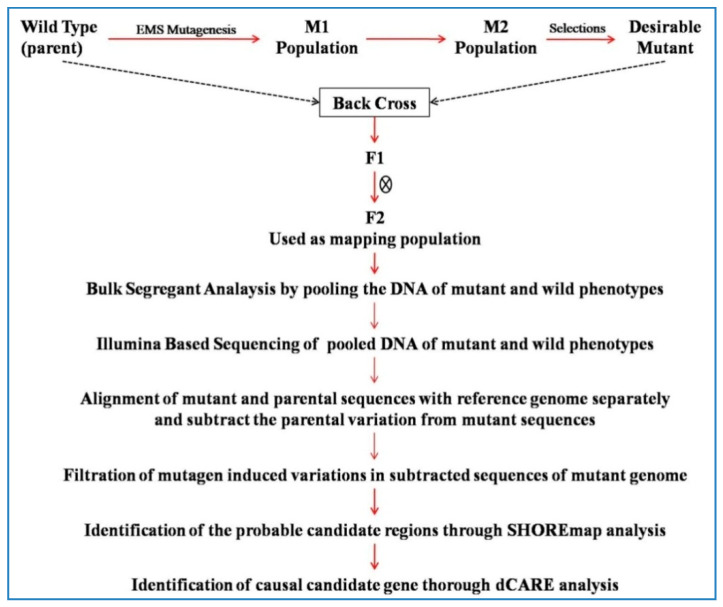
Flow diagram showing the major steps of deep CAndidate RE-sequencing (dCARE) approach of forward genetic screen.

Hartwig et al. [14] proposed deep candidate resequencing (dCARE) that combined isogenic bulk sequencing with deep candidate genes resequencing to identify and map the causal mutations in the Arabidopsis genome. They have demonstrated the use of mutagen induced variation as segregating markers in mapping-by-sequencing for identification of candidate gene/causal mutagens. dCARE is a NGS based Ion Torrent Personal Genome Machine sequencing platform for identification and mapping of causal mutations. The assumption being made in this technique was that the highest frequency of the causative change occurs in pools of bulked segregants among all EMS-induced changes (Figure 3). If only resequencing is used then it will not possible to distinguish between the subtle allele frequencies that are closely linked in EMS changes [14]. dCARE has potential to identify the subtle phenotypes that were previously inaccessible. dCARE provides increased coverage for linked changes that reduced the large number of candidate genes to single causal gene.

In the dCARE technique, sequencing of bulked DNA from F_2_ population may be performed to identify the putative mutations or hot spot regions based on allele frequencies. Thereafter, the actual candidate mutations are identified through new Ion Torrent sequencing technology. This is a WGS based approach to characterize mutants in model as well as in non-model crop species. Using such a technique, Hartwig et al. [14] unequivocally identified mutation in At3g63270 that corresponded with the suppressor mutation like in Arabidopsis [14].

### 6.4. MutMap Approach

**Figure 4:**
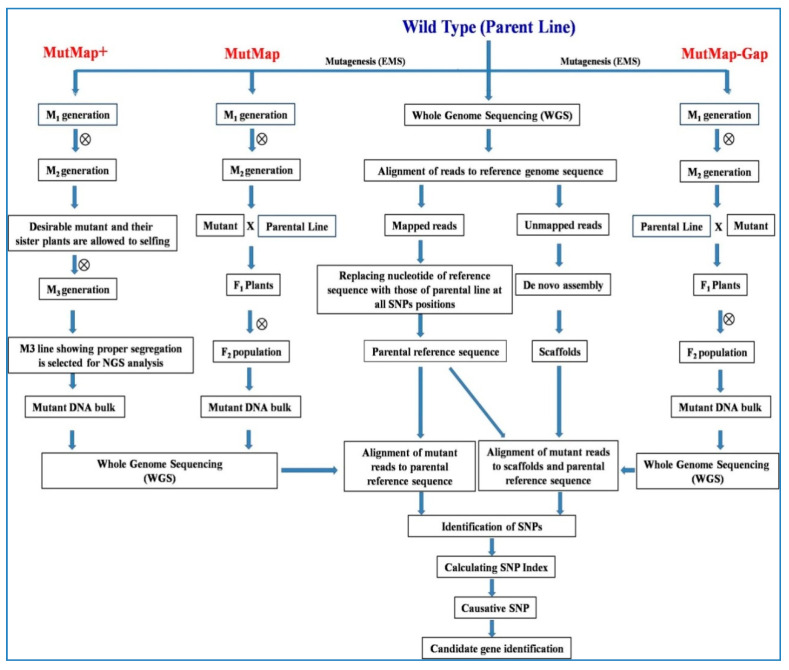
Flow diagram showing the major steps of MutMap, MutMap+c, and MutMap-Gap approaches of forward genetic screen.

MutMap is based on whole genome sequencing of bulked DNA from mutant progenies to identify the causal mutations for the trait of interest [13]. In MutMap process, a mutant having altered phenotype identified in the M_2_ or later generation is crossed to the wild type parent. The resultant F_1_ plants are allowed to self-pollinate and F_2_ population is screened for segregating mutant and wild type phenotype. DNA from multiple F_2_individuals (about 20–30 individuals) showing the mutant and wild phenotype are pooled and subjected to NGS based WGS with substantial genomic coverage (<10×). Simultaneously, a parental reference sequence is constructed by re-sequencing the wild type parent and aligning the reads to the publicly available reference genome of the species (Figure 4). In the consensus sequence, nucleotides of the reference sequence are replaced with those of parental line at all the detected SNP positions to make the parental reference sequence. The F_2_ bulked sequence reads are then aligned to this parental reference sequence and result of the alignment is used to infer the genomic location of the causal SNPs responsible for the mutant phenotype. The majority of the SNPs that are unlinked or loosely linked to mutant phenotype will segregate in a 1:1 mutant/wild type ratio, but SNPs which are linked to mutant phenotype will show 0% wild-type and 100% mutant reads. Calculation of SNP index, which is the ratio between the number of reads of a mutant SNP and the total number of reads corresponding to the SNP helps to predict the linkage of loci to the mutant phenotype. The SNP index of 1 or near to 1 indicates that SNP is linked to mutant phenotype, whereas those near to 0.5 correspond to the unlinked loci. MutMap can detect all kind of nucleotide variations created by mutagenesis such as SNPs, insertion and deletions.

Abe et al. [13] for the first time demonstrated the MutMap by characterization of two mutant rice genotypes (Hit1917-pl1 and Hit0813-pl2) having pale-green leaf phenotypes. They have successfully identified a causative SNP in the chlorophyllidee*a*-oxygenase (*OsCAO1*) gene that lead to a L253F mutation (codon CTT→TTT) resulting in pale-green phenotype. Similarly, Takagi et al. [47] used MutMap to characterize a salt tolerant rice mutant (*hst1*) of rice cultivar Hitomebore and identified a SNP in the third exon of the *OsRR22*gene as the causative mutation. This SNP caused a nonsense mutation (codon TGG → TAG) in *OsRR22*gene and was linked with the salinity-tolerance phenotype of *hst1*mutant. Authors also developed a salt tolerant rice variety “Kaijin” by backcrossing the mutant *hst1* to parental line Hitomebore for two generations and confirmed the presence of the mutant *hst1*allele through Sanger sequencing. Variety “Kaijin” took only two years to develop and was practically similar to cultivar Hitomebore, except for the *hst1*mutation. This demonstrated the power of genomics-based crop breeding approaches for accelerating the development of climate ready improved cultivars. Chen et al. [120] mapped a novel nuclear male sterility mutant (*ms9*) in sorghum using MutMap and identified the causal mutation for male sterility in *Sobic.002G221000*gene (designated as *Ms9*), which encode a plant homeotic domain (PHD)-finger transcription factor critical for tapetum degeneration and pollen formation. The *Ms9* gene was the first nuclear male sterility gene identified in sorghum and provided an opportunity to control male sterility for the development of a two-line breeding system for hybrid sorghum. MutMap has been used for identifying agronomically important genes in many crop plants such as rice, sorghum, soybean, wheat, maize, etc. (Table 1).

### 6.5. MutMap+ Approach

MutMap+ method is an emerging technique which could be better exploited to identify mutated allele and causal SNPs. Unlike MutMap, if mutants obtained here are lethal or sterile, it is difficult to make crosses and thus cannot be used to identify the causative genes, therefore MutMap+ has been developed to deal with this setback [15]. MutMap+ is an NGS based approach which identifies genetic variations in wild and mutant strain simultaneously by whole genome sequencing. Showing similarity with the other genetic mapping methods, it also uses the principle of genetic linkage [90]. It follows the principle of mapping by sequencing through Bulk Segregant Analysis [15].

In MutMap+, after treating the seeds with desired mutagen, they are sown to obtain M_1_ plants. Mother panicle/ear of the M_1_ plants are harvested and are sown in panicle/ear to row method in the desired season to generate M_2_ population. Mutants found in M_2_ population are tagged. Sister plants (plants present in the row in which mutant is obtained) of the mutant are also tagged. Seeds from the mutant as well as each sister plant is harvested separately and are sown in panicle to row method in next season to constitute M_3_ generation. Here, in M_3_ some of the sister plants are observed to exhibit segregation into mutant and wild type plants. A total of 20 to 40 M_3_ plants showing the mutated characters and 20 to 40 M_3_ plants showing the wild-type characters are selected for DNA isolation and bulked to form two groups/bulks. With NGS machine (for e.g., Illumina GAIIx sequencer), sequencing of these two bulked samples is done. To detect the SNPs, the obtained sequence is aligned to the reference genome and SNP index is generated. Frequency of SNPs is used to create SNP index which is the ratio of the number of sequence reads that have a mutant SNP to the total number of the sequence reads corresponding to the SNP. This index showing indicates its close proximity with the causal gene and 0.5 depicts the location of SNPs away from the genomic region. The whole genome is scanned to detect the SNP index. Genomic region indicating SNP index 1 is the potential region possessing causal mutation site (Figure 4) [15].

MutMap+ is applied to find the causal mutation site and identify the gene/allele responsible for the trait occurred due to mutation. Nakata et al. [127] screened a mutant population of a japonica cultivar Nipponbare and found two mutant lines with altered starch gelatinization property. MutMap+ discovered that both of these lines harbor novel mutant alleles (age alleles) of starch branching enzyme IIb (BEIIb) gene. Since MutMap+ involves genetic mapping without artificial crossing, it can be exploited for rapid gene identification in many crops where crossing is a difficult choice. MutMap+ describes rapid identification of genes/QTL from natural variants or mutants using NGS.

### 6.6. MutMap-Gap

One of the main requisite of MutMap is that the presence of causative mutation in the parental reference sequence and therefore, MutMap cannot identify mutations located in the gap region of the reference genome. To overcome this shortfall, Takagi et al. [16] proposed a modified approach called MutMap-Gap for the identification of causal mutations in the gap regions of reference sequence. MutMap-Gap is a combination of MutMap and targeted de novo assembly of genomic gap regions. In MutMap-Gap, the parental reference sequence is prepared by re-sequencing the parental line and aligning the resulting reads to publicly available reference genome as in MutMap [13]. The majority of sequence reads from parental line align with the reference genome. However, when the parental line displayed significant genetic variations from the reference genome, the reads derived from a parental line specific genomic region cannot be aligned to the reference genome and therefore, remained unmapped (Figure 4). Therefore, mutations present in these gap regions (unmapped reads) cannot be identified by MutMap, but approximate position of the causative mutation can be delineated by MutMap-Gap method.

Takagi et al. [16] demonstrated MutMap-Gap to isolate the blast resistant gene *Pii* from the rice cultivar Hitomebore, using a mutant line that has lost *Pii* function. The mutation was located in the gap region of the reference genome of rice cultivar Nipponbare and therefore causative SNPs cannot be identified by MutMap. Using MutMap Gap analysis, a causative SNP was identified in the second exon of the gene *Os09t0327600-01*, which was located in the gap region. This SNP represented a nonsense mutation, causing an amino acid change from Tryptophan (TGG) to a stop codon (TGA) and lead to the mutant phenotype. The MutMap-Gap is a good approach for identification of novel gene in cultivars which are genetically distinct from reference genome.

### 6.7. RNA Sequencing (RNA Seq) Based Mapping

RNA sequencing-based approach is useful in identifying and mapping the genomic regions/candidate genes harboring a mutation and appropriate lesions causing altered phenotype in a strain [17]. This approach may be applied in the model organism/plants whose genome is already sequenced and genome sequences are available. In the RNA-seq the sample being sequenced is limited to the expressed portion of the genome/genes. Hence, it reduces the large amount of sequence data and effectively identifies possible mutations, causing nonsense and mis-sense changes, affecting splicing, and affecting gene expression [17].

Benefit of the RNA-seq based approach is to directly identify and assess the consequences of splice-altering mutations [17]. RNA-seq approach of NGS mapping offers numerous benefits over the WGS as it minimizes the representation of the genome to the expressed portion thus reduce amount of sequencing data and its cost. The effect of candidate mutation can be directly assessed in mutants and expression level in the expressed genes. It facilitates the identification of a very small number of high priority nonsense and mis-sense candidates underlying a phenotype of interest. It has potential to enhance the efficiency of forward genetics screens in model systems with large, polymorphic genomes [145].

RNA-seq based approach have been successfully applied for development of D-genome specific chromosomal marker in synthetic hexaploid wheat [141] and identification of stem resistance locus in *Aegilops umbellulata* [140]. It can also be applied to better understanding of the molecular mechanisms and genetic consequences domestication of crop plants [145].

### 6.8. QTL-Seq Approach

**Figure 5:**
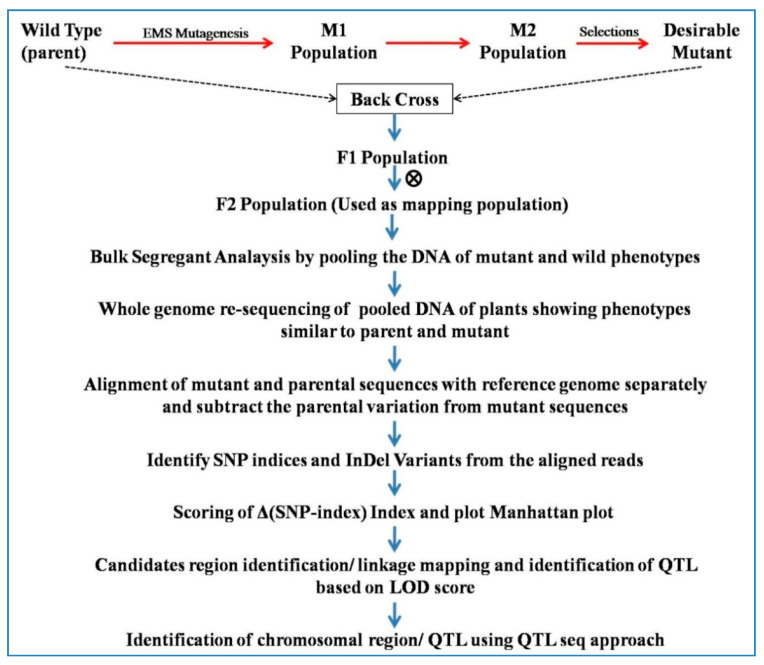
Flow diagram showing the major steps of quantitative trait loci (QTL) seq approach.

Most of the agronomically important traits are controlled by multiple genes having individual minor effects which are known as quantitative trait loci (QTLs). Identification and mapping of desired QTLs by whole-genome resequencing (WGR) of DNAs from two extreme populations for a given phenotype is called QTL seq approach [18]. In order to map QTL using QTL-seq approach, mapping populations which are generated from two genotypes having contrasting phenotypes for desired traits is required. The double haploid (DH) and Recombinant inbred lines (RILs) populations showed a high degree of homozygosity and are suitable for identification of QTLs of minor effects. In other cases, BC1F_2_ progenies (10–20 individuals) showing two discrete characters may be selected for DNA isolation from both the groups and isolated DNA may be pooled for further process through QTL-seq approach [61,146]. DNA bulks are subjected to WGR with a minimum coverage of >6× genome and sequenced short reads are aligned to the reference genome to estimate the SNP-index. It is expected that the bulked DNA contains 1:1 ratio of genomes from both parents in the majority of genomic regions. Unequal representation of the two parental genomes containing QTLs for desired trait may also be detected. The short reads are aligned with the reference genome and the numbers (k) of short reads harbouring SNPs that are different from the reference sequence are counted. The QTL can be identified as peaks or valleys of the SNP-index plot. Accordingly, the SNP- index is 0 if the entire short reads contain genomic fragments from the parent that was used as a reference sequence. The SNP-index is 1 if all the short reads represent the genome from the other parent. A SNP-index of 0.5 means an equal contribution of both parents’ genomes to the bulked progeny (Figure 5).

QTL-seq allows an accurate quantitative evaluation of the genomic contribution from the two parents to the bulked DNAs by using SNP-index. QTL-seq does not require DNA marker development and genotyping and thus offers the rapid QTL identification with much higher power than the previous methods. It can be applied to any population for detecting genomic regions that underwent artificial or natural selection.

The QTL-seq approach has been successfully applied in rice [18], cucumber [136], tomato [147], chickpea [135,148], peanut [149], watermelon [150,151], broccoli [152,153], and squash [139] for identification and mapping of QTLs/genes.

### 6.9. Exome Capture Approach

Mapping by traditional positional or map-based cloning is being gradually replaced with mapping by sequencing approaches. Numerous approaches and successes have been demonstrated in Arabidopsis and some other plant models using approaches like SHOREmap and NGM. However, despite tremendous success of NGS based mapping in such systems, the extension of the same methods to complex genomes like wheat and barley has not been successful, due to very high amounts of repetitive regions and polyploidy in these genomes. Genomes of these important crops are very complex and hence, genomic data analysis and fishing of gene of interest is very cumbersome [132]. An alternative to whole genome analysis is the analysis of only expressed part of the genome or the exome. Traditionally exome includes all protein coding exons, small RNA and some additional locus with known function. In exome capture the exome region is fished out using hybridization with biotinylated probes which binds to target regions. The sample is the amplified and used for NGS or long-range sequencing approach. This reduction in complexity from entire genome to only coding region greatly helps in accelerating mapping of target genes (Figure 2). For example, wheat genome is 17 Gb in size, whereas the exome is only 84 Mb, and similarly, barley genome is 5 Gb in size, the exome is much smaller (62 Mb) [154]. This has led to identification of many important genes in both the crops like identification of “*MANY-NODED DWARF*” gene responsible for dwarfness in X-ray induced deletion in barley, *Yr6*yellow rust resistance gene in wheat, and several others [132,155].

Exome sequencing has been extensively used in forward genetics screening and cataloguing of mutant locus [20,156,157]. Simultaneously, this method is also useful in reverse genetics screening. Using exome capture in tetra and hexaploidy wheat with 84 Mb capture probes, over 10 million high confidence point mutations in coding regions of an EMS induced Targeting Induced Local Lesions in genome (TILLING) population were identified. The average density of mutations was observed to be 35–40 mutation per kb, roughly equivalent to 24 non-synonymous mutations per annotated wheat gene [155]. The method also identified large scale deletion mutants in 29% of wheat lines. Similar approach identified causal mutation for tall mutant in wheat to present in *Rht-B1*gene, which is known in plant height phenotype [132].

### 6.10. NIKS (Needle in the k-Stack) Approach

Most of the NGS based forward genetic approaches are based on the availability of reference genome for comparison, which restricted the use of these techniques on the organisms where genetic map is available or genome has been sequenced. To overcome this problem, NIKS (needle in the *k*-stack) has been developed by Nordstrom et al. [21] and experimentally validated its applicability in rice cultivars and *Arabis alpina*. In both species, they found similar mutations while comparing the pooled F_2_ individuals as well as in M3 individuals. Therefore, they concluded that NIKS may be applied for forward genetic screen in any species (where mutagenesis is amenable) without requiring segregating populations, genetic maps, and reference sequences. NIKS is a forward genetic approach for reference-free genome comparison, discovery of homozygous mutations, gene identification, and mapping based exclusively on the frequencies of *k-mers* (a set of short subsequences) within the WGS data of two closely related genomes, such as mutant and wild-type genomes [21].

To identify the candidate mutations by excluding/reducing the undesirable variations, mutant genotype is crossed with their wild parent and genome of F_2_ individuals are used for WGS [13,14]. Thus NIKS utilizes the bulked segregant pooling based WGS strategy to identify the unknown or novel mutations in the genome. However, in species where crossing is difficult, two allelic mutants of their M_3_ seedlings may be utilized for the same purpose through NIKS.

**Figure 6:**
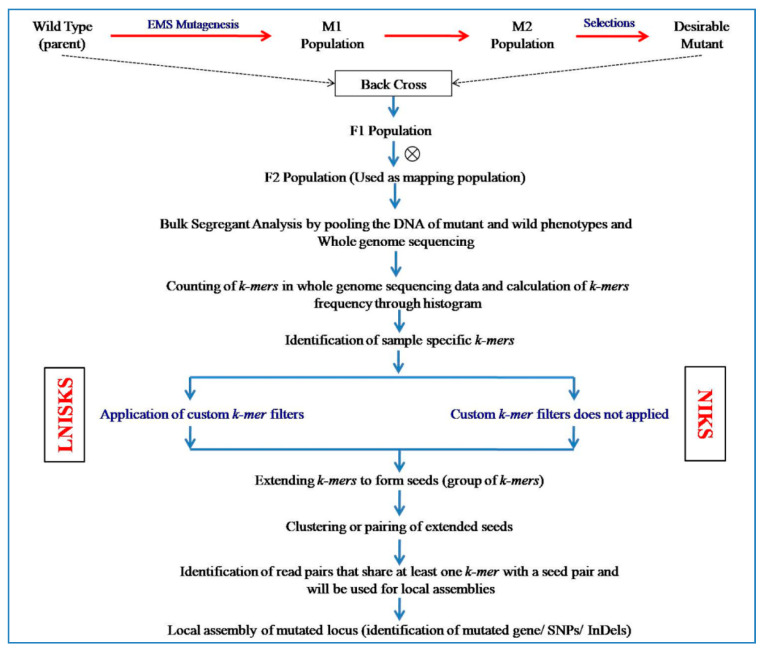
Flow diagram showing the major steps of reference genome independent approaches, needle in the k-stack (NIKS), and longer needle in a scanter k-stack (LNISKS).

At the first step of NIKS, frequency of each *k-mers* maybe estimated within the WGS data of each sample using *k*-*mers* counting software Jellyfish [158]. Native *k-mers* and *k-mers* overlapping with sequencing errors can be distinguished by *k-mers* frequency histograms. Therefore, *k-mers* overlapped with sequencing errors may be separated easily from the reads that are error free. To reduce the sequencing errors raised during amplification artifacts, filtering for identical *k-mers* may be performed before running the NIKS. Differences in genome sequences of two populations generate numerous specific and overlapping *k*-*mers* (Figure 6).

NIKS technique firstly identifies the sample specific *k-mers* and merges them to form long sequences or seeds. Differences in seeds of both populations may arise due to induced mutagenesis. NIKS considers only those seeds which are homologous but not identical (obtained through pairing of seeds of both populations i.e., wild-type and the mutant population seed). Small mutations (less than *k* − 1 bp) and small InDels are combined in one elongated seed whereas larger InDels might not be assembled into one seed.

In the last step, NIKS generates local de novoassemblies or contigs to extend the sequences associated with the mutated region in the genome. Furthermore, de novogene predictions or gene annotation alignments may be used in the generated contigs for functional analysis of putative causal mutations. Based on these, accurate seed pair that represents the homozygous mutagen induced changes has been identified and determined. A major advantage of NIKS algorithm is that it does not require segregating populations, genetic maps and reference sequences for bioinformatics analysis; it has potential to identify the mutations within repetitive regions in some extent; it is useful in development of markers/SNPs/InDels in non-model organisms with reference-independent methods [21].

### 6.11. MutChromSeq (Mutant Chromosome Sequencing) Approach

DNA sequencing based mutational genomics techniques are very costly and compel significant computational challenges in some important crops such as barley, rye, and wheat. Genome size of these crops is huge (in GBs) and highly complexed polyploid nature. Therefore, traditional map based cloning has been applied for cloning of only a limited number of their genes [159]. Advancement of NGS techniques enabled several new gene cloning approaches viz., methylation filtration [160], duplex-specific nuclease digestion [161], transcriptome sequencing [162], and exome capture sequencing [20]. These approaches reduce the DNA sequence complexity and sequencing costs but they are unable identify all the potentially significant sequences.

To avoid these complications, the MutChromSeq (Mutant Chromosome Sequencing) approach was proposed by Sanchez-Martin et al. [22] for rapid gene isolation in barley and wheat. MutChromSeq is a chromosome flow sorting and sequencing based powerful, sequence-unbiased and reference-free forward genetics approach for genome complexity reduction and induced causal mutations identification without having positional fine mapping [163]. It has been effectively applied to reclone the *Eceriferum-q* gene (resistant to wax covered leaf sheath); *Rph1* (leaf rust resistance gene) in barley [130] and clone the *Pm2* gene (Powdery mildew resistance) in wheat [22].

**Figure 7:**
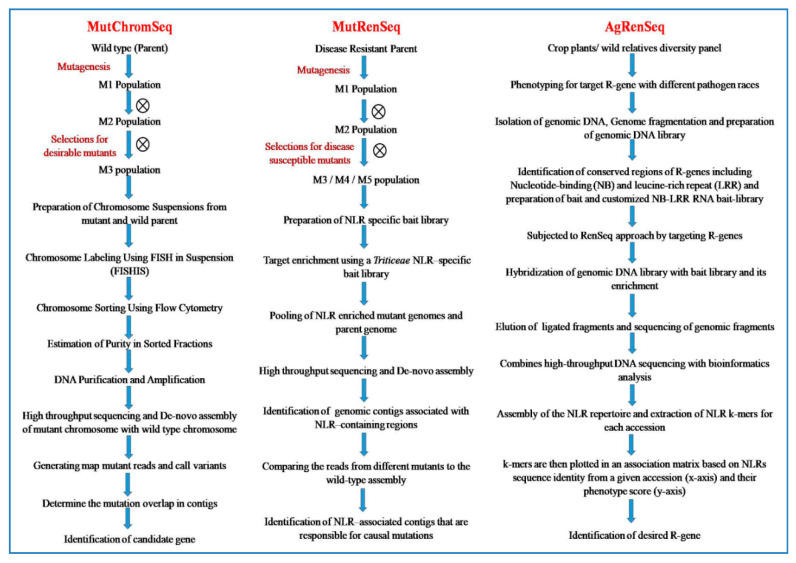
Flow diagram showing the major steps of MutChromSeq, MutRenSeq, and AgRenSeq approaches.

It does not depend on recombination or fine-mapping for gene cloning and causal mutation identification. This approach may be performed in any crop species where, the mutagenesis is feasible; the target gene is associated with a phenotype and the chromosomal location of target gene is known [22,163]. Chromosomal sequence comparison of multiple independently derived mutants and their wild parents confirms the identification of causal mutations in a single candidate gene or a non-coding sequence. MutChromSeq does not follow the recombination-based mapping and targets all the DNA sequences and is therefore very useful for forward genetic screening in crop species that have complex genomes. Methodology of MutChromSeq approach is briefly presented in flow diagram (Figure 7).

### 6.12. MutRenSeq Approach

MutRenSeq is a resistance gene (R-gene) cloning pipeline, which integrates the EMS based mutational genomics with exome capture targeting R-genes to identify the causal mutations in a single candidate gene. It defines the mutagenesis of R genes prior to cloning and identification with target capture sequencing. It was developed by Steuernagel et al. [23] to clone the stem rust resistance genes *Sr22* and *Sr45* from hexaploid bread wheat. MutRenSeq is the advanced version of R-gene enrichment sequencing (RenSeq) approach [164]. This method utilizes the RenSeq data of EMS derived loss-of-resistance mutants (disease susceptible mutant) with wild-parent to compare the R-gene complements for identification of R-gene/causal mutations. This method enabled the fast identification of disease resistance genes (R-gene) without any positional cloning or fine mapping [163]. This approach was further utilized by Marchal et al. [131] for cloning of three major and distinct genes (*Yr7, Yr5* and *YrSP*) for yellow rust resistance in wheat (*Triticum aestivum* L.). They avowed that the nucleotide binding and leucine-rich repeats (NLRs) encoding genes may provide diverse resistance spectra to important fungal diseases [131]. MutRenSeq is particularly appropriate for plant species with large genome size (wheat, barley, rye) where whole genome sequencing of multiple individuals is complicated and expensive [23].

MutRenSeq works on the principle of exome capture targeted to the R-gene complement (NLR sequence) and mutational genomics. It generates a wild type RenSeq data based de novo assembly to be utilized as reference to map the RenSeq data of susceptible mutants [163]. Therefore, MutRenSeq includes the generation, screening, and identification of disease susceptible mutants or loss-of-function mutants from the M_2_ population of disease resistant line followed by RenSeq. In order to identify the susceptible mutants, EMS mutagenesis should be done in the genotype in which resistance is controlled by a single R-gene. Because the susceptible or loss-of-function mutant may generate only when mutation occurs directly in the R-gene. A genotype harboring two or more R-genes for disease resistance would not be appropriate for MutRenSeq because there may be difficulties to select the susceptible mutants [23].

MutRenSeq requires three major steps for fast isolation of resistance genes viz., (i) Identification and selection of disease susceptible mutant (loss of disease resistance) from resistant wild type parent, (ii) Sequencing of NLR enriched genomes of both susceptible mutant and wild type resistant plants (iii) comparing these genes in mutants and wild types to identify the exact mutations responsible for the loss of disease resistance. Methodology of MutRenSeq approach is briefly presented in flow diagram (Figure 7).

### 6.13. SIMM (Simultaneous Identification of Multiple Causal Mutations)

Simultaneous Identifications of Multiple Mutations (SIMM) was invented to identify causal mutations in multiple mutations at a time by analyzing simultaneously their sequence data. The method does not demand wild type parental genome sequence information for the analysis. Here, each novel mutant (obtained from same mutagenized population) was back-crossed with the parent, and DNA of 20–30 F_2_ individuals having phenotype of mutant were pooled (for each mutant different mutant pools should be obtained) and sequenced to >20× of the genome size using NGS method. Clean reads from each mutant-pool sample were then aligned to the reference genome (available for the crop) using software SOAP2 [165], bwa [166], or Bowtie2 [167]. Exclusively mapped reads were retained for SNP calling using SOAPsnp (for SOAP2) [168] or SAM tools (for bwa and Bowtie2) [143]. Total SNPs available in each bulk of discrete mutant may be identified and compared with each other to pointing out the candidate SNPs for each mutant phenotype. Moreover, to reduce sequencing error and increase the precision, few SNPs having <5 supporting reads were excluded from the further analysis. To overcome the problem of SNP index in MutMap or other techniques, Allele Index (AI) was introduced to consider SNPs supporting wild-type alleles in background mutants. AI may be calculated by dividing the number of supporting reads from wild-parent to the total number of reads in the mutant genotypes. AI with 0.8 value represents the availability of 20% sequencing errors in the particular SNPs. Moreover, candidate SNPs may be further refined by applying the Euclidean Distance (ED) analysis [169] to consider the SNP index in the test strain and reference mutants. ED value was calculated as *ED =* [2 (SI_t_ − Si_bc_)]^1/2^, where SI_t_ stands for the SI of mutation allele in the test strain, whereas SI_bc_ stands for the SI of the same mutation allele in the background mutants. Since ED value ranged from 0 to 2^½^, it was raised to power 6 (ED^6^), which was promising enough to enlarge the differences between causal mutations and closely linked mutations, and to signify candidate regions. Candidate regions harboring the causal mutation were expected to show a cluster of SNPs with high SI and ED^6^ values. Finally the candidate mutations were validated using phenotype association study through high resolution melting analysis [170].

The main advantages of SIMM are identification of candidate mutations in several mutants simultaneously, originated from a single wild parent; no need of the reference sequence of parental genotype and assembly of sample specific *k-mers*; exclusion of a large number of background polymorphisms through the use of other mutant’s data; avoidance of wrongly retaining or excluding candidate sites; high precision for resolving the causal mutations; applicability to detect the allelic relations among mutants with similar phenotypes.

The founding research paper [24] had characterized seven mutants obtained from EMS mutagenesis of Huanghuazhan (HHZ) rice. Of the seven mutants, four were male sterile. The study revealed mutations in three different genes (*LOC_Os04g39470*, *LOC_Os03g58600*, *LOC_Os07g32480*) responsible for male sterility in rice. Later, one such mutant HT5763 which showed mutations in *LOC_Os04g39470* was deeply characterized to reveal the molecular mechanism for male sterility in rice. The locus *LOC_Os04g39470* codes for OsMyb80 gene where a G to A mutation in HT5763 caused Glu74 (GAG) substitution by Lys (AAG). The mutations affects the activity of *OsMyb80* and this compromised mutated Myb80 was not able to promote the gene expression of downstream genes that synthesize precursors for pollen wall formation; transportation of small nutrient molecules to nurture the pollen cell growth; degradation of the cell wall surrounding pollen mother cells (PMCs) and the tetrads for microspore separation; massive protein degradation, redox homeostasis, and cell death gene expression associated with the tapetum; and signal transduction and transcriptional regulation that regulate downstream events for pollen development [171].

### 6.14. TACCA (Targeted Chromosome-Based Cloning via Long-Range Assembly)

NGS based approaches rely upon amplification of target sequence and massive parallel sequencing, which resulted in high depth of sequencing and coverage of genome. However, NGS approach suffers in region of high repetitive sequences or structural elements like transposons. Due to very short read length (20–30 bp) of NGS approach, it is almost impossible to align millions of repeat short reads to a reference genome or de novo assembly sequence [22]. This is a particular challenge in crops like wheat and barley which have a very high percentage of the genome as repetitive elements.

To overcome the short read length limitation of second-generation sequencing approaches, a few alternative technologies providing long read lengths have been utilized, termed third generation sequencing. Unlike second-generation technologies the third generation sequencing does not relies on amplification of target sequence, instead sequences a single DNA molecule. The sequence is generated in real-time and read lengths are in average of the order of 12–15 kb with claimed lengths of up to 100 kb. The long read lengths of this technology remove the hurdles of computational algorithms for genome assembly, and hence are very useful for denovogenome assembly, transcript assembly, and mapping mutations [22]. Although long range sequencing is believed to accelerate functional genomics, it has a limitation of accuracy of prediction of bases in sequence and very high cost compared to short read sequencing. In general as the read length increases in sequencing the read quality decreases, which then require intervention either in experiment design or computational approach to deal with accuracy. The error rates of third-generation long range sequencing is higher than second-generation short read sequencing, which may be critical in mutant identification, causal SNP identification studies. Several computational approaches have been devised to overcome the error rate issue; however, it still remains a challenge for long range sequencing.

**Figure 8:**
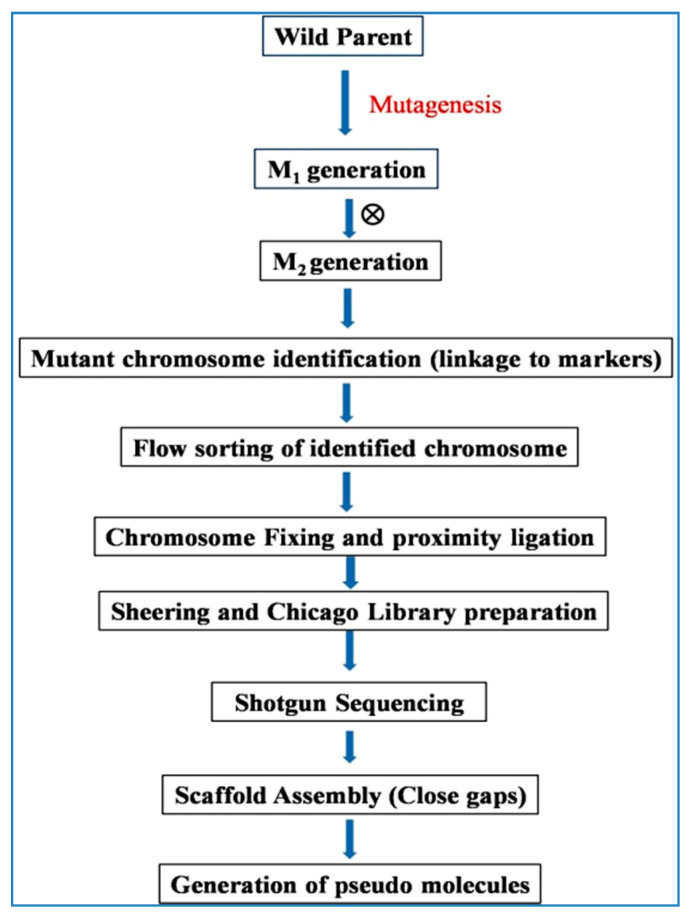
Flow diagram showing the major steps of TACCA (Targeted chromosome-based cloning via Long-range assembly) approach of forward genetic screen.

Currently, two major technologies viz. Pacific Biosciences Single Molecule Real Time (SMRT) sequencing and Oxford Nanopore’s technology are utilized for long range sequencing. A combination of short and long read approaches termed hybrid sequencing is proposed for best results of both the techniques. This approach relies on fixing of DNA in the nucleus before sequencing using various approaches, followed by standard WGS. As a result the sequences which are closer together will have higher read pairs compared to sequences which are far apart. Long reads from third generation approaches may be used to create long template or scaffolds or sequencing repeat regions and then short read from second generation approaches may be used to remove the small errors from this sequence [22]. Targeted chromosome-based cloning via long-range assembly (TACCA), utilizes sequencing of isolated desired single chromosome separated by flow cytometry using long range sequencing approach (Figure 8) [22,25].

TACCA approach was used to clone wheat gene *Lr22a* which is an important leaf rust resistance gene transferred to the bread wheat from wild relative *Aegilops taushchii*. *Lr22a* was mapped on chromosome 2D using microsatellite markers, further with high resolution mapping a 0.48-cM interval on map flanked by two markers was delimited. 2D chromosome from *Lr22a* carrier and non-carrier genotypes were isolated using flow cytometry and subjected to long range sequencing and also short read sequencing to alleviate errors. The long range sequencing using this approach lead to scaffolds with 50% of them having sizes 9.76 Mb or more with highest scaffold up to 36.4 Mb which is around 100× more than a BAC library. Using combination of short read sequencing and mutant analysis the *Lr22a*locus was positively identified as NLR enriched R-gene [25]. Target Enrichment Sequencing (TenSeq) approaches have been recently reported to be very useful in cloning of target genes in complex genomes like wheat. These approaches like MutRenSeq, MutChromSeq, AgRenSeq are dependent on NGS based sequencing and assembly. Long range sequencing in such cases especially where repeat regions are encountered is highly useful for cloning of target genes. TACCA approach has many advantages over traditional map based cloning approach viz., no dependence on enrichment library and reference sequence. In addition, this approach is fast and cost effective compared to BAC based cloning [25].

### 6.15. AgRenSeq (Association Genetics with R-Gene Enrichment Sequencing) Approach

AgRenSeq is the techniques that allows cheaper discovery and cloning of resistance (R) gene from a diverse germplasm panel and wild relatives of any crop species [26]. This technique does not require reference-genome and can directly identify the nucleotide-binding/leucine-rich (NLR) regions which confer resistance rather than identifying a genomic region encoding multiple paralogs. Screening of wild plants for variety of diseases and sequencing them can be done to identify resistance genes. Association analysis was combined with RenSeq approach to screen and identify the R-gene, for RenSeq a bait library that targets R-genes in particular plant species is required. A sequence capture bait library was designed and optimized for capturing nucleotide-binding/leucine-rich (NLR) sequences encoded by the R-genes in this population. The enriched R-gene sequences were then assembled into NLR contigs and NLR k-mers were extracted for each accession. After a pre-filtering step, *k-mer* based association mapping was conducted to identify *k-mers* associated with the resistance trait. Phenotype scores are converted to AgRenSeq scores that assign positive values to resistance and negative values to susceptibility. Intermediate phenotype should have an AgRenSeq score close to zero (Figure 7). It was successfully applied in wheat crop and four stem rust resistance genes; *Sr33*, *Sr45*, *Sr46*, and *SrTA1662*, against three races of the stem rust pathogen were identified using this approach [26].

Subsequently, this approach was recommended for rapid cloning of R-gene and facilitates marker-assisted breeding and broad-spectrum resistance engineering in genetically modified crops without a need for a reference genome [172]. It also serves interrogate pan-genome sequence variation in diverse germplasm to isolate uncharacterised R-genes. Recent examples have shown utility of RenSeq for improving disease resistance in plants, and similar techniques identification of genes for abiotic stress-tolerant will greatly benefits the crops [173]. AgRenSeq exploits entire gene set of all strains of a species to isolateand cloning of the uncharacterized R-genes. However, biasness during NLR capture may mislead the isolation and cloning of R-gene through AgRenSeq approach [26].

### 6.16. LNISKS (Longer Needle in a Scanter K-Stack) Approach

LNISKS, an extension of NIKS, is a high throughput method for mutation discovery and mapping in crop genome, especially in large and repetitive crop genomes without availability of reference genome. This follows the similar principle of NIKS except for applying the custom *k-mer* filter to increase the precision of identification of causative mutations (Figure 6). Other innovations in LNISKS are pertaining to extension of *k-mers* to seeds both before and after the seeds are clustered/paired [27]. Suchecki et al. [27] experimentally proved and validated that the filtering of *k-mers* significantly reduces the quantity of call variants which has to be taken in WGS data of 16Australian wheat cultivars. This method was used for identification and mapping of *ms5* genic male sterility mutations in bread wheat. Furthermore, LNISKS also recognized the markers which are responsible for narrowing the *Ms5/ms5* genomic region.

In LNISKS, once sample-specific *k-mers* are identified, a new customized *k-mer* filtering step is applied based on availability of suitable data and specific biological context of the input datasets [27]. This step relieves the computational requirements by significantly reducing the total number of *k-mers* taken for the final assembly and also reduces the number of false positive calls by removing the irrelevant loci. This helps in identification of desired candidate mutations in the genome. Higher *k*-values in combination with limited sequencing coverage increased the proportion of target sequences, not being captured by *k-mers*. Therefore, fixing the *k-value* at or slightly below of that value is appropriate for further analysis. The main advantages of LNISKS are simultaneous identification of homozygous as well as heterozygous mutation; reference and alignment-free genotyping of second generation sequencing datasets for a pre-defined set of varying loci; applicability for mutation discovery and mapping in complex and large genomes like wheat (17 Gb) and reduces the computational cost and time over NIKS [27].

## 7. Bioinformatics Tools/Software/Pipelines Used in NGS Based Forward Genetic Screen for Mutation Identification and Mapping

Bioinformatics are essential for processing and analyzing the large and complex genomic datasets and obtaining their functional insights. The advent of next-generation sequencing (NGS) has drastically changed the process of associating a phenotype with their causal gene/QTL with the help of sophisticated bioinformatics tools [28,174]. NGS based forward genetic screening may possess high cost and the complexity of analyzing high throughput sequencing data. Assembly of sequenced plant genome is severely hampered by long repetitive segments, large genome sizes, and polyploidy genome [7,8,175]. Advances in sequencing technologies and bioinformatics tools have allowed rapid progress since the sequencing and assembly of the crop genome [7]. Various bioinformatics tools/pipelines such as SHOREmap [11]; MAQGene [29]; GenomeMapper [30]; Mapping and Assembly with Short Sequences (MASS) [31]; Next-Generation Mapping (NGM) [12]; The SNPtrack tool [32]; CloudMap [33]; CandiSNP [34]; SIMPLE Pipeline [35]; artMAP [28] have been developed for reducing the complexity of NGS data and delivering the concluding outcomes (Table 4). These pipelines/tools are further highlighted with their importance and applicability in subsequent heads.

### 7.1. MAQGene

MAQGene is a user-friendly, simple web browser interface developed by Bigelow et al. [29] especially to detect the causative mutations and to further classify the mutations based on associated exon annotations in *Caenorhabditis elegans*.

MAQGene automatically launches the publicly available MAQ (Mapping and Assembly with Quality) software and collects the customized summary and functional outputs (viz., position and specific features of sequence variants) from the WGS data of mutant genome compared to a wild-type reference genome. It does not require any technical knowledge of command-line tools and can be run through the web interface entirely. The MAQGene have specific set of parameters for analyzing and interpreting WGS reads. The user may choose the appropriate set of parameters according to need. MAQGene may handle long reads (up to 127 bases) and map in both single read and paired-end modes. The output file of MAQGene is easily convertible to an Excel spreadsheet for further processing. Various measures of output files allows user to perform easy browsing of sequence variants; comparisons of different genomes; fast assess the degree of coverage for a given nucleotide position; selection of desirable variants for validation by Sanger re-sequencing and the test probing true and functional relevance of a nucleotide variant.

Bigelow et al. [29] used MAQGene for discovering sequence variants generated by in-house Illumina Genome Analyzer-II based WGS reads in different *C. elegans* genomes compared to the wild-type *C. elegans* reference genome. In addition, it may also useful to compare any input WGS reads (in fastq format) to any wild-type reference genome (fastq format with general-feature format). MAQGene has been broadly used by the scientists while working with *C. elegans* [87,176]. However, it was no longer updated by the developers because the pipeline relies on an outdated aligner (MAQ) and requires technical expertise to install, which inevitably limits its general adoption.

### 7.2. GenomeMapper

GenomeMapper is a standalone algorithm to simultaneously align the short reads of multiple genomes by integrating related genomes into a single graph structure [30]. It provides accurate and high alignment quality by aligning a sequence against a graph of sequences rather than aligning two linear sequences. This algorithm firstly provided the standards to tackle the problems arising from aligning the multiple references. It was specific tool for the Arabidopsis 1001 Genomes Project [177] and provides incessantly basic short read alignment option for pipeline SHORE [178]. Schneebergeret al. [30] demonstrated the construction of a multiple genome sequence graph based on published polymorphisms of Arabidopsis and compared the results with the conventional approach of aligning the same set of reads against a single reference. However, GenomeMapper may be used to analyze sequence reads obtained from bacterial, plant, invertebrate, and mammalian genomes.

Moreover, GenomeMapper can also be used for alignments against a single target genome and provides access to regions that are highly divergent from the first reference. This approach reduces the number of false-positive SNP calls caused by misalignments near InDels [178]. Furthermore, it is useful tool for precise alignment of longer reads of whole genome sequence of known species or related species and single step analysis of metagenomic samples. GenomeMapper broadly follows the three steps. In the first step, GenomeMapper scans the hash index for *k-mers* which are identical between sequence reads and genome graph. In the second step, location and sequence of Nearly Identical Maximal Substrings (NIMS) between sequence reads and genome graph is being determined. At last, a k-banded alignment by applying dynamic programming to ensure a consistent gap placement is being executed.

### 7.3. MASS (Mapping and Assembly with Short Sequences)

MASS (Mapping and Assembly with Short Sequences) was created by Cuperus et al. [31] from the Carrington Laboratory, specifically for mapping and assembling of sequenced data of Arabidopsis mutants. It is freely available for download and utilization at http://jcclab.science.ore-gonstate.edu/MASS. MASS pipeline is utilized to identify the small number of candidate genes/causative mutations within a relatively small interval of 1–2 Mb from the DNA sequence of bulk segregant population. MASS package have potentials for simultaneous mapping and sequencing at a genome-wide level. It employs Illumina sequenced pair end reads obtained from DNA pools of BC_1_F_2_ populations of mutant and wild parent for mapping process.

Additionally MASS package may be used to identify the *mir390a-1* mutation in *Arabidopsis thaliana*. It includes pipelines to create SNP enrichment plots, alignment with MAQ, and SNP filtering. The MASS pipeline contains preloaded scripts to run Cache Assisted Hash Search using XOR logic (CASHX) [179], Short Oligonucleotide Analysis Package (SOAP) [180] and Mapping and Assembly with Quality (MAQ) [29] for its various functioning. MASS utilizes CASHX to perform mapping process illumine sequenced reads whereas SOAP is utilized to define the syntactic roles of aligned Illumina reads. MASS filters the SNP data set (cns.snp) from the MAQ output assembled them into short intervals. Cuperus et al. [31] nicely explained the application of MASS for identification of MIR390 mutants in Arabidopsis by direct genome sequencing. They informed that MASS is extremely useful for causal mutation identification in EMS derived mutant genome, where hundreds or thousands of changes existed in addition to the causal mutation.

### 7.4. Next-Generation Mapping (NGM)

The NGM approach can be accessed on web at http://www.bar.utoronto.ca/NGM/index.html. The application can be used for mapping of EMS based mutants in Arabidopsis. The experimental setup involves creating a mapping population (F_2_) from mutant and wild type parents, NGS sequencing of pooled genomic DNA of mapping population, aligning the resultant sequence with reference genome, and identification of SNP. This output SNP data is submitted to the web based interface for NGM [12]. The web-based system works in the following four step process:

i. SNP data from F_2_ mapping population: This involves getting sequence data from sequencer, cleaning, and pre-processing of sequence data. Uploading and filtering of SNP data to website.
ii. Localization of SNPs: Localization of mutants to Arabidopsis chromosome is done by identifying non-recombinant (less heterozygosity) area within genomic region with mutations.
iii. Segregating SNPs based on their variation to reference genome.
iv. Localization and annotation of causal SNP by fine mapped region.

The NGM approach relies on two key parameter values for narrowing down on putative locus. These parameters viz. kernel size (used for smoothing of chastity threads) and number of cluster used for *k*-means clustering, are essential to be used selected optimally, and may influence the mutant locus identification power. A bigger kernel size provides greater smoothing of chastity threads losing on potential SNPs while a smaller kernel size although improve resolution may lead to potential errors. The *k* parameter in *k*-means clustering is essential for separation of homozygous and heterozygous signals. Large *k-*values increase homozygous to heterozygous ratio but may not correspond to actual size of candidate region [12].

### 7.5. The SNPTrack Tool

Leshchiner et al. (32) developed the SNPTrack tool by calculating the log likelihood based on a Hidden Markov Model of recombination breakpoints to make possible the rapid and accurate identification and mapping of causal mutations in model organisms. They have used the SNPTrack tool for analysis of sequencing data of zebrafish. SNPTrack adopts a client–server system that integrates data management, analysis, and interpretation into a single system [32].

SNPTrack was developed as a one-stop-shop bioinformatics solution capable of performing the functional analysis of for genetic data. This tool offers a full suite of data storage and management, analysis, and interpretation tools for genetic association studies [181,182]. The Oracle server stores and integrates phenotypic and genotypic data as well as annotations of genetic biomarkers from public resources about SNPs, quantitative trait loci (QTLs), genes, proteins, and pathways. SNPtrack was used to analyze the data and determine the causative mutation [32] and further it was applied to the study the epigenetic control of intestinal barrier function and inflammation in zebrafish [183] and role of MYB36 to regulate the transition from proliferation to differentiation in the Arabidopsis root [184].

### 7.6. CloudMap

CloudMap is an open source cloud computing resource for mapping of mutants, originally designed for *C. elegans* but applicable to Arabidopsis and other plants. The CloudMap is available originally on galaxy web platform (http://www.usegalaxy.org/cloudmap) which has been shifted to MiModD system (http://mimodd.readthedocs.io/en/latest/). Cloud map may be run on galaxy cloud or amazon web service platform or a local instance on machine [33]. CloudMap uses custom python scripts for mutant locus identification from NGS based reads. CloudMap provides following features; (i) alignment, variant calling, and annotation; (ii) variant subtraction and filtration; (iii) checking of candidate genes for mutation and creating useful gene lists; (iv) in-silico complementation testing; and (v) identification of deletions.

### 7.7. CandiSNP

CandiSNP is a web based and user-friendly bioinformatics application to identify the causal mutations/SNPs from high throughput sequencing (HTS) data of F_2_progenies having mutants and parental phenotypes. It was developed by Etherington et al. [34] to enables fast assessment of causal SNPs and their positions. CandiSNPcreates density plots from the HTS data provided by the user, therefore identification of SNP positions is essential before CandiSNP activity.

CandiSNP perform two important steps viz., use of snpEff [185] to categorize the SNPs based on chromosomal position and chromosome wise plotting the SNPs into a graph based on user-selected alternate allele frequency (AF) threshold, provides the desired information by coloring SNPs in different densities according to SNP categories. The density and distribution of SNPs is visualized chromosome wise in the output line graph to decipher the causal SNPs [34].

Candidate causative mutations/SNPs in annotated coding regions are highlighted on the plots/graph and listed in a table. Furthermore, CandiSNP gives annotations describing the genomic feature in which each SNP is located. This function is very useful in creating associations between SNPs/causal genomic regions and their molecular and biological functions. Based on this, selection and refinement of candidate gene becomes easier and faster. CandiSNP is useful in identification of recessive mutants in homozygous F_2_ (BC_1_F_2_) segregants generated from a back-cross as well as dominant mutations in homozygous F_2_ (BC_1_F_2_) after confirming their homozygosity in the F_3_ prior to bulk segregant analysis. By plotting homozygous and close-to homozygous SNPs identified from HTS along the chromosome arms, the program visualizes areas of linkage and easily narrows down candidate mutation positions [34].

The web-application CandiSNP is freely available online at http://candisnp.tsl.ac.uk. User may run the CandiSNP process on a command line as part of bioinformatics pipelines, a Perl module is also available as part of the source code. At present, CandiSNP is available for the genome annotations of several plant species viz., *Arabidopsis thaliana* TAIR9 and TAIR10, *Oryzae sativa* v7, *Solanum lycopersicum* v2.40, *Glycine max* 1.09v8, *Vitus vinefera* v1, and *Zea mays* B73 v5b. This web application has been utilized by Wambugu et al. [186] and Xu et al. [187] to dissect the genetic control of amylose content and study the Lincomycin (LIN)-mediated inhibition of protein synthesis in chloroplastsin rice, respectively.

### 7.8. A SIMPLE Pipeline

SIMPLE pipeline (Simple Mapping Pipeline) is a NGS based user friendly and easy to use bioinformatics tool to identify the causal mutations in forward genetic screens. It was developed by Wachsman et al. [35] to identify and map the causal mutations in a simple and easy way. This pipeline utilizes the NGS fastq reads; generated from WGS of DNA pool of mutant type and wild type progenies to create resultant tables and plots which have information about all the possible candidate genes and causal SNPs. The pipeline is based on a short BASH script in order to generate several variant call format files and three plots. The program may be operated on Mac OSX version 10.11.6 and Linux release 6.7 (GNOME 2.28.2) with having Java 1.7 installed on the system. SIMPLE pipeline is hosted on GitHub and may be easily downloaded from https://github.com/wacguy/Simple and installed without any difficulty. It may be operated without prior understanding of NGS programming and bioinformatics tools. It requires only a few simple preparatory steps *viz.*, downloading the fastq files and determining the species to start the program.

Wachsman et al. [35] suggested that the pipeline may work with any paired end or single-endfastq combination obtained from M_2_/F_2_ or M_3_/F_3_. However, working with an F_2_/M_2_ generation rather than an F_3_/M_3_ generation is more fruitful to obtain robust outcomes. It does not require NGS reads from any map cross or back cross [13,15]. An important consideration is the sequencing depth; pipeline prefers sequencing depth up to 30× to get reliable results. Additionally, this pipeline may work with fewer numbers of sampled individuals (a few dozen) which further shortens the analytical complexity and analysis time. The SIMPLE pipeline can be useful for analysis of any diploid species; however it has only been validated for *Arabidopsis thaliana* and *Oryza sativa* (rice) [35].

### 7.9. artMAP

artMAP, a user-friendly tool, was developed to discover and map EMS induced mutations in the Arabidopsis genome [28]. The artMAP may be operated on Windows/Mac/Linuxoperatingsystemplatforms and its pipelines consist of several open source software integrated into a docker container (https://www.docker.com/) to provide a graphical user interface (GUI). This software has overcome the limitations of data generation platforms and it allows the data generated by all the sequencing platforms. Input sequencing files generated from single or paired-end sequencing can be used by artMAP for the mapping of EMS induced mutations in plants. By artMAP, mapping and identification of causal mutations may be possible with only a few mouse clicks and analysis results may come out with interactive graphs which display the annotation details of each mutation [28].

Due to its graphical user interface (GUI) artMAP can be run on a standard desktop/laptop, thereby limiting the bioinformatics expertise required. The artMAP pipeline consists of well-established tools including TrimGalore, BWA, BED Tools, SAM tools, and SnpEff which were integrated in a Docker container. The artMAP pipeline consists of six steps performed by integrated softwares, namely (i) pre-processing of the sequencing read files by Trimgalore; (ii) alignment of reads to the Arabidopsis genome by BWA; (iii) post-processing of aligned reads by SAMtools (iv) identification of single-nucleotide polymorphisms (SNPs) specific to mutant samples through the combined use of SAMtools and BED Tools suite, (v) Visualization of the SNPs, annotation of SNPs by SnpEff and; (v) Finally, artMAP provides a list of SNPs along with their allele frequency, depth, and annotation in a tab separated file. artMAP also provides an additional option to control the removal of PCR duplicates from the control and mutant BAM files. This bioinformatics tool helps to map EMS-induced mutations in Arabidopsis and asses their association with the desired phenotype [28].

## 8. Limitations and Way Ahead

Next-generation sequencing platforms have enhanced our knowledge in sequencing and mapping of crop genomes, identifying causal mutations, gene regulation, and more [181]. NGS based techniques are gaining great achievements in functional genomics, agri-genomics, and plant breeding research and have prospects for their utilization in other potential fields of research in the future [188].

**Figure 9:**
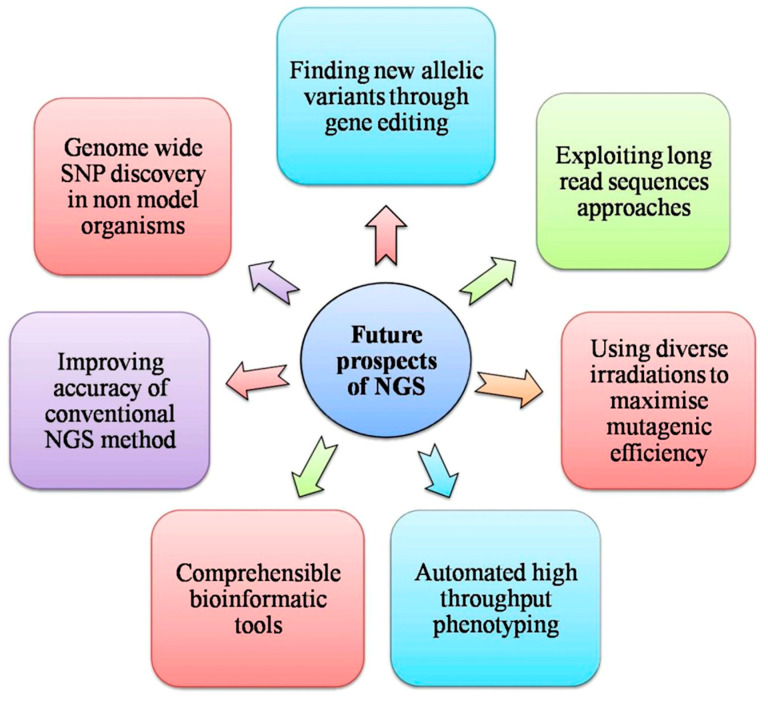
Future prospects of next generation sequencing for crop improvement.

It is well known that most of the plant genomes are complex with relatively higher proportion of the repetitive sequences and transposons. Due to this, the short sequence reads (35–700 bp) generated by second generation sequencing platforms viz. Illumina, SOLiD, Roche (454) etc. are sometime not efficient, especially if the genome size is big [100,189]. Long read sequencing (~several Kb) produced by third generation sequencing platforms like Oxford Nanopore and PacBio appears to be more promising. This will enable identification of epigenetic marks such as DNA methylation in highly variable genomic regions and its expression. It is also helpful in constructing high quality reference genome and accelerate gene discovery in plants [109,190]. With the help of NGS techniques, genome wide SNP discovery, allele mining, developing molecular markers and genotyping can be performed in other species or on non-model organisms, facilitating its speedy use in research activities [93]. However, the detection of rare point mutations in plant genome through NGS remains challenging. To improve the accuracy of conventional NGS method, Stalhberg et al. [191,192] developed an improved version of sequencing called as Simple, Multiplxed, PCR-based bar-coding of DNA for selective mutation detection using sequencing (SiMSen-Seq). It can detect variants at or below 0.1% frequency with low DNA input. Similarly, to overcome the limitations of mutation detection in reduced representation sequencing, Monson-Miller et al. [193] demonstrated the use of Restriction Enzyme Sequence Comparative ANalysis (RESCAN) to detect single nucleotide polymorphism (SNP) in rice and *Arabidopsis*. Though the genotyping/sequencing has made significant progress in the last decade, phenotyping did not register a similar pace. Automated high throughput phenotyping platforms for greenhouse as well as field can definitely accelerate the gene discovery even further (Figure 9) [190].

Future perspective of NGS in plant breeding is obtaining new allelic variants in the genome of many crops. NGS technologies have made available genome sequences for many important crops which will facilitate genome editing approaches. These editing approaches enable site directed mutagenesis to improve economically useful traits by involving modification in targeted locus. Sequence-specific nucleases, such as zinc finger nucleases (ZFN), transcription activator-like effector nucleases (TALEN) and CRISPR/Cas9 system can be exploited for the same (Figure 9) [194]. Using diverse irradiation methods to generate mutants and their characterization by refined NGS pipelines would become more popular in future studies. Combining irradiation facilities like heavy ion beams, cosmic rays, etc., and DNA sequencing technologies will maximize mutagenesis efficiencies and will optimize the use of the developed genetic materials for plant breeding and functional genomics investigations [190,191,192].

## 9. Conclusions

It is worth to say that mutation breeding became one of the major pillars of modern plant breeding as it plays an important role in global nutritional and food security now. With the advent of molecular marker techniques, mapping and cloning techniques, next generation sequencing approaches and functional genomics, induced mutagenesis has become more useful and feasible for crop improvement as well as for discovering the novel candidate genes and their biological functions. The NGS techniques have made the mapping and sequencing procedures more feasible and became an essential tool for crop geneticists to identify and characterize genomic variations associated with economically important traits. WGR and transcriptome profiling, which contribute to providing comprehensive information on genetic variability and its regulatory mechanisms, are the most popular applications of NGS. The NGS-based approaches presented throughout this review are applicable to classic mutations whose phenotypes fall into distinct categories compared to wild type (qualitative traits). Moreover, many of them are applicable even in the absence of a reference genome, known single-nucleotide polymorphisms, or genetic tools. With these advancements, rapid identification and mapping of desired mutations are now possible in forward genetic screens. Additionally, these approaches also provide a wealth of background mutations in germplasm collections that carry the mutations to the scientific community. However, to be more successful in the interpretation of NGS data, bioinformatics and statistical tools are essential for delivering accurate assembly, alignment, and variant detection. Therefore, a modern plant breeding team must have the scientists from multidisciplinary background viz., plant biology, genetics, physiology, molecular biology, bioinformatics, statistics, and mathematics to ensure the precision and success of research work in this direction. Furthermore, the advent of third-generation sequencing technology, associated with longer reads, will further improve the quality of variant and mutation identification. We do hope that the information provided in this review will be useful for all the scientific communities working on these aspects.

## Figures and Tables

**Table 1:** Crop wise and country wise status of crop mutants in the world

S. No.	Top 10 Countries Who Has Developed the Highest Number of Crop Mutants		Top 10 Crops Having Highest Number of Crop Mutants	
	Country	Number of Mutants	Crops	Number of Mutants
1	China	810	Rice	833
2	Japan	479	Barley	305
3	India	341	Chrysanthemum	285
4	Russian Federation	216	Wheat	264
5	The Netherlands	176	Soybean	175
6	Germany	171	Maize	89
7	United States	139	Groundnut	78
8	Bulgaria	76	Rose	67
9	Bangladesh	75	Common bean	57
10	Viet Nam	58	Cotton	48

Source: FAO/IAEA Mutant Variety Database [39].

**Table 2:** Application of next-generation sequencing (NGS) based forward genetic approaches for identification and mapping of mutated gene in various crops.

S. No.	NGS Based Techniques/Approaches	Name of the Gene(s)	Trait(s)	Crop/Species	Strategy Followed	Population Used	Sequencing Platform	Depth (×)	References
1	MutMap	*OsCAO1*	Pale green leaf	Rice (*Oryza sativa* L.)	Mapping by sequencing through BSA	BC_1_F_2_	Whole-genome sequencing using an Illumina GAIIx sequencer	>12×	[13]
2	MutMap	*OsRR22*	Salt tolerance	Rice (*Oryza sativa* L.)	Mapping by sequencing through BSA	BC_1_F_2_	Whole-genome sequencing using Illumina GAIIx or Illumina HiSeq2500	-	[44]
3	MutMap	*Os04t0413500* *(* *WB1* *)*	White-belly endosperm	Rice (*Oryza sativa* L.)	Mapping by sequencing through BSA	BC_1_F _2:3_	Whole-genome sequencing using Illumina HiSeq2500	30×	[108]
4	MutMap	*08SG2/OsBAK1*	Small grain (sg2)	Rice (*Oryza sativa* L.)	Mapping by sequencing through BSA	BC_1_F_2_	Whole-genome sequencing	-	[109]
5	MutMap	*OsEDR1* gene	Spotted-leaf mutants (spl101 and spl102)	Rice (*Oryza sativa* L.)	Mapping by sequencing through BSA	BC_1_F_2_	Whole-genome sequencing	-	[110]
6	MutMap	*LOC_Os06g29380*	Yellow leaf and dwarf 1 (*yld1*)	Rice (*Oryza sativa* L.)	Mapping by sequencing through BSA	BC_1_F_2_	Whole-genome sequencing	-	[111]
7	MutMap	*DEP2-1388*	Erect panicle (R1338)	Rice (*Oryza sativa* L.)	Mapping by sequencing through BSA	BC_1_F_2_	Whole-genome sequencing	-	[112]
8	MutMap	*OsNRAMP5*	Low Cadmium accumulation (lcd1)	Rice (*Oryza sativa* L.)	Mapping by sequencing through BSA	BC_1_F_2_	Whole-genome sequencing using Illumina HiSeq4000	-	[113]
9	MutMap	*ent-kaurene oxidase 1 (OsKO1)*	Delayed seed germination	Rice (*Oryza sativa* L.)	Mapping by sequencing through BSA	BC_1_F_2_	Whole-genome sequencing using Illumina HiSeq 2000	-	[114]
10	MutMap	*OsCADT1*	Enhanced cadmium tolerance and selenium enriched grain	Rice (*Oryza sativa* L.)	Mapping by sequencing through BSA	BC_1_F_2_	Whole-genome sequencing using Illumina HiSeq4000	40×	[115]
11	MutMap	*Os05G0312000*	Spotted-leaf mutant (spl40)	Rice (*Oryza sativa* L.)	Mapping by sequencing through BSA	BC_1_F_2_	High throughput sequencing	25×	[116]
12	MutMap	*OsRLCK109* (LMM24)	Lesion mimic	Rice (*Oryza sativa* L.)	Mapping by sequencing through BSA	BC_1_F_2_	Whole-genome sequencing using Illumina HiSeq	50×	[117]
13	MutMap	SUPERNUMERARY BRACT(SNB)	Loss of shattering	Rice (*Oryza sativa* L.)	Mapping by sequencing through BSA	BC_1_F_2_	Whole-genome sequencing using llumina HiSeq2500	-	[118]
14	MutMap	*MS1*	Male Sterility 1 (Ms1)	Wheat (*Triticum aestivum* L.)	Mapping by sequencing through BSA	BC_1_F_2_	Whole-genome sequencing using Illumina HiSeq 2500	-	[119]
15	MutMap	Sobic.002G221000 (*Ms9*)	Nuclear male sterility	Sorghum (*Sorghum bicolor*)	Mapping by sequencing through BSA	BC_1_F_2_	Whole-genome sequencing using Illumina X-10	15×	[120]
16	MutMap	Sobic.001G228100 (GDSL-like lipase/acylhydrolase)	Devoid of epi-cuticular wax (EW)	Sorghum (*Sorghum bicolor*)	Mapping by sequencing through BSA	BC_1_F_2_	Whole-genome sequencing using Illumina X-10	15×	[121]
17	MutMap	*Bna.IAA7.C05*	Dwarfism	Oilseed rape (*Brassica napus*)	Mapping by sequencing through BSA	BC_1_F_2_	Whole-genome sequencing using Illumina HiSeq X 10	-	[122]
18	MutMap	*ZmCLE7*	Fasciated-ear mutant	Maize (*Zea mays* L.)	Mapping by sequencing through BSA	BC_1_F_2_	Whole-genome sequencing using Illumina HiSeq platform	20×	[123]
19	MutMap	Zm00001d028818 (*Dek1*)	Very narrow sheath (vns)	Maize (*Zea mays* L.)	Mapping by sequencing through BSA	BC_1_F_2_	Whole-genome sequencing using Illumina Hi-Seq 2500	-	[124]
20	MutMap	*Glyma.04g242300*	Spotted leaf-1 (spl-1)	Soybean (*Glycine max* L.)	Mapping by sequencing through BSA	BC_1_F_2_	Whole-genome sequencing using Illumina HiSeq 2500	-	[125]
21	MutMap Gap	*Os09t0327600-01* (*Pii*-1)	Susceptibility to rice blast fungus (M. Oryzae)	Rice (*Oryza sativa* L.)	Mapping by sequencing through BSA	BC_1_F_2_	Whole-genome sequencing using Illumina GAIIx sequencer	-	[16]
22	MutMap Gap	NLR gene (*Pii-2*)	Susceptibility to rice blast fungus (M. Oryzae)	Rice (*Oryza sativa* L.)	Mapping by sequencing through BSA	BC_1_F_2_	Whole-genome sequencing using Illumina NextSeq500	-	[126]
23	MutMap+	*SNP variants*	Leaf colouration	Rice (*Oryza sativa* L.)	Mapping by sequencing through BSA	M_3_ population	Whole-genome sequencing using an Illumina GAIIx sequencer	-	[15]
24	MutMap+	Starch branching enzyme IIb (*BEIIb*) gene	Starch gelatinization property	Rice (*Oryza sativa* L.)	Mapping by sequencing through BSA	M_3_ population	Illumina Whole-genome sequencing sequencer	>10×	[127]
25	MutMap+	CqCYP76AD1-1	Green hypocotyl mutant (*ghy*)	*Chenopodium quinoa*	Mapping by sequencing through BSA	M_3_ population	Illumina Whole-genome sequencing	-	[128]
26	MutMap+	OsLAP6/OsPKS1	Sterility	Rice (*Oryza sativa* L.)	Mapping by sequencing through BSA	M_3_ population	Illumina Whole-genome sequencing	-	[129]
27	MutChromSeq	*Eceriferum-q*	Resistant to wax covered leaf sheath	Barley (*Hordeum vulgare* L.)	Gene cloning by chromosome flow sorting and sequencing	M_3_ population	Illumina HiSeq2000 platform	27×	[22]
28	MutChromSeq	*Rph1*	Leaf rust resistance	Barley (*Hordeum vulgare* L.)	Gene cloning by chromosome flow sorting and sequencing and mutational genomics	DH, RIL and M_4_ Population	Illumina HiSeq in rapid run mode	18–30×	[130]
29	MutChromSeq	*Pm2*	Powdery mildew resistance	Wheat (*Triticum aestivum* L.)	Gene cloning by chromosome flow sorting and sequencing and mutational genomics	M_3_ population	Illumina HiSeq2000 platform	35×	[22]
30	MutRenSeq	*Sr22* and *Sr45*	Stem rust resistance	Wheat (*Triticum aestivum* L.)	Exome sequencing of R-gene complements (NB-LRR sequence) and mutational genomics	M_3_ population	Illumina MiSeq or HiSeq platforms at TGAC	-	[23]
31	MutRenSeq	*Yr7*, *Yr5* and *YrSP*	Yellow rust resistance	Wheat (*Triticum aestivum* L.)	Exome sequencing of R-gene complements (BED-NLR sequence) and mutational genomics	M_5_ population	Illumina MiSeq or HiSeq platforms	-	[131]
32	NIKS (needle in the k-stack)	*OsCAO1 gene*	Pale green leaves and semidwarfism	Rice (*Oryza sativa* L.)	Identification of frequencies of short subsequences (*k-mers*) within WGS data of two populations	M_3_ population	Illumina MiSeq or HiSeq platforms	51× and 105×	[21]
33	NIKS (needle in the k-stack)	*floral defective 1* (*fde1*)	Floral homeotic defects	*Arabis alpina*	Identification of frequencies of short subsequences (*k-mers*) within WGS data of two populations	M_3_ population	Illumina MiSeq or HiSeq platforms	51× and 105×	[21]
34	LNISKS (longer needle in a scanter k-stack)	*ms5 gene*	Genic male sterility	Wheat (*Triticum aestivum* L.)	Identification of frequencies of short subsequences (*k-mers*) within WGS data of two populations with custom k-filter	BC_1_F_2_	Illumina HiSeq2500 platform	19× and 23×	[27]
35	TACCA (Targeted chromosome based via Long-range assembly)	*Lr22a*	Leaf rust resistance	Wheat (*Triticum aestivum* L.)	Chromosome sorting, followed by long range chromosome sequencing.Proximity ligation of in-vitroreconstituted chromatin (Chicago)	F_2_ for mapping and M_2_ for further validation.	Illumina HiSeq 2500	30×	[25]
36	NGM (Next Generation Mapping)	*fph-1*, *fph-2*, *mur11-1*	Flupoxam hypersensitive, MURUS-11 both genes involved in cell wall composition.	Arabidopsis (*Arabidopsis thaliana*)	Short read sequencing of F_2_ bulks followed by SNP identification in regions of low recombination.	F_2_ bulks	Illumina GA IIx	30× or higher	[12]
37	Exome Capture	*Rht-B1*	Height in wheat plants.	Tetraploid Wheat (*Triticum turgidum*)	The whole complement of exons (coding regions) can be enriched and sequenced using an exome capture approach to reduce the number of bases sequenced leading to lower assay costs.	M_4_ or stable mutants	Illumina HiSeq 3000	20×	[132]
38	Exome Capture	*Chimeric allele of Lr21*	Leaf and Yellow rust	Hexaploid Wheat (*Triticum aestivum* L.)	The whole complement of exons (coding regions) can be enriched and sequenced using an exome capture approach.	M_4_ or stable mutants.	Illumina HiSeq 2000	>20×	[133]
39	Exome Capture	*SNP variants*	-	Barley and Wheat (*Triticum aestivum* L.)	Target sequences derived from full-length cDNA or RNA-Seq contigs are aligned against the Morex assembly.	Wild types and improved cultivars	Single HiSeq2000 lane	20×	[19]
40	Exome Capture	*SNP variants*	-	Allotetraploid Wheat (*Triticum turgidum*)	Target sequences derived from full-length cDNA or RNA-Seq contigs are aligned against the Morex assembly.	Wild types and improved cultivars	Single HiSeq2000 lane	20×	[134]
41	QTL-Seq	*Nortai qPi-nor1(t) qPHS3-2*	Partial resistance to the fungal rice blast disease and seedling vigor	Rice (*Oryza sativa* L.)	QTL identification by combining bulked-segregant analysis and whole-genome resequencing	RIL and F_2_	Illumina Genome Analyzer IIx	>6×	[18]
42	QTL-Seq	*SW QTL* (*CaqSW1.1*)	100-seed weight QTL	Chickpea (*Cicer arietinum* L.)	NGS-based whole-genome QTL-seq strategy	F_4_ mapping population	Illumina HiSeq2000 Sequencer	91–93% coverage	[135]
43	QTL-seq	*Ef1.1*	Early flowering QTL	Cucumber (*Cucumis sativus* L.)	NGS-based whole-genome QTL-seq strategy	F_2_ and BC_1_F_2_	Illumina Genome Analyzer IIx machine	8×	[136]
44	QTL-seq	*qTGW5.3*	Grain size and weight	Rice (*Oryza sativa* L.)	Bulk segregant analysis and whole genome resequencing	F_2_ (NIL-F_2_)	HiSeqXTen (Illumina Sequencer)	30×	[137]
	QTL-seq	*Glyma.13 g249400*	Plant Height	Soybean (*Glycine max* L.)	Bulk segregant analysis and whole genome resequencing	F_2_ and F _2:3_	Illumina HiSeqPE150 machine.	10×	[138]
45	QTL-seq	Chr 4 (*QtlPC-C04*), 11 *QtlPC-C11*) and 14 (*QtlPC-C14*)	Resistance to Phytophthora crown rot in squash	Squash (*Cucurbita moschata*)	QTL-seq bulk segregant analysis	F_2_ population	Illumina HiSeq X Sequencer	45×	[139]
46	RNA-seq (BSR-Seq)	*hoxb1bb1219*, *nhsl1bfh131*, *vangl2m209*, *egr2bfh227*	-	Zebrafish *Xenopustropicalis*	RNA sequencing based bulked segregant analysis	BC_1_F_2_	Illumina HiSeq 2000 machine	-	[17]
47	RNA-seq (BSR-Seq)	QTL detected for races TTTTF and TTKSK	Stem resistance locus in *Aegilops umbellulata*	Asiatic grass (*Aegilops umbellulata*)	RNA sequencing based bulked segregant analysis	F_2_, bi- parental mapping populations	Illumina HiSeq 2500	-	[140]
48	RNA-seq based BSA (BSR-Seq)	*Net2 gene*	Synthetic wheat	Wheat (*Triticum aestivum* L.)	RNA sequencing-based bulked segregant analysis	bi-parental mapping population	Illumina MiSeq sequencer	-	[141]
49	SHOREmap	*AT4G35090*	Slow growth light green leaves	Arabidopsis (*Arabidopsis thaliana*)	Mapping by sequencing through BSA	BC_1_F_2_	Illumina Whole-genome sequencing	22×	[11]
50	deep CAndidateREsequencing (dCARE)	*Heterochromatin protein1 (lhp1)*	Chromatin-mediated gene repression	Arabidopsis (*Arabidopsis thaliana*)	Mapping by sequencing through BSA	BC_1_F_2_	Illumina Whole-genome sequencing	41×	[14]
51	Simultaneous Identification of Multiple Causal Mutations (SIMM)	*LOC_Os03g43670 (H-224 mutant) LOC_Os03g58600 (H-190 mutant)*	Open hull and brownish palea/lemma Male sterility	Rice (*Oryza sativa* L.)	SIMM simultaneous analyze the multiple mutants derived from the same parental plants, with no parental reference genome. It follows Mapping by sequencing through BSA approach.	BC_1_F_2_	Whole-genome sequencing at Illumina Hiseq 2000 platform	>20×	[24]
52	AgRenSeq	*Sr33*, *Sr45*, *Sr46* and *SrTA1662*	Stem rust resistance	Wheat (*Triticum aestivum* L.)	AgRenSeq exploits entire gene set of all strains of a species to isolate the uncharacterized R-genes	Germplasm lines	Illumina short-read sequencing	-	[26]

**Table 3:** Comparisons among the various NGS based forward genetic approaches.

S. No.	NGS Based Technique/Approach	Principle	Population Required	Reference Genome Required (Yes/No)	Applicability/Scope (All Species or Any Specific)	Firstly Demonstrated by
1	MutMap	Mapping by Whole Genome Sequencing through BSA	BC_1_F_2_	Yes	Applicable in all where crossing is possible and reference genome is available	[13]
2	MutMap Gap	Mapping by sequencing through BSA	BC_1_F_2_	Yes	Applicable in all where crossing is possible and reference genome is available	[16]
3	MutMap+	Mapping by Sequencing through BSA	M_3_	Yes	Applicable for the mutants where crossing is difficult or the traits which appear early	[15]
4	MutChromSeq	Gene cloning by chromosome flow sorting and sequencing and mutational genomics	M_3_ population	No	Applicable to wheat, barley, rye and other crop species where mutagenesis is possible	[22]
5	MutRenSeq	Exome sequencing of R-gene complements (NB-LRR sequence) and mutational genomics	M_2_ /M_3_/M_4_/M_5_	No	Applicable to plant species with large genome size (wheat, barley, rye) where mutagenesis is possible	[23]
6	NIKS (needle in the k-stack)	Estimation of the frequencies of *k-mers* (short subsequences) on the WGS data of two highly related genomes	M_3_	No	Applicable to all organisms. However, especially useful for non-model organism where genome has not been sequenced and where mutagenesis is feasible.	[21]
7	LNISKS (longer needle in a scanter k-stack)	Estimation of the frequencies of *k-mers* (short subsequences) on the WGS data of two highly related genomes with custom k-filters.	BC_1_F_2_ or F_2_	No	Applicable to all organisms. However, especially useful for complex genomes and large and repetitive crop genomes like wheat (17 Gbp).	[27]
8	TACCA (Targeted chromosome based via Long-range assembly)	Generation of a long range scaffold of chromosome with help of either chromosome contact map method or proximity ligation of in-vitroreconstituted chromatin (Chicago).	BC_1_F_2_ or M_2_ lines	No	Applicable to all crop species.	[25]
9	NGM (Next Generation Mapping)	Identification of causal mutation using sequencing of F2 bulks and computational short downing to SNP present in genomic region of low recombination.	BC_1_F_2_	Yes	Applicable to all species with good quality reference genome.	[12]
10	Exome capture	Mapping of traits only in the expressed portion of genome, to avoid complexities due to size, repetitive elements etc. present in genome.	BC_1_F_2_ bulk or M_2_ lines.	No	Applicable to all species, but more powerful in sequenced genomes.	[19]
11	QTL-Seq	QTL-seq combines bulked-segregant analysis and whole-genome resequencing	BC_1_F_2_, RIL and DH	Yes	Applicable to all species where whole genome sequence and mapping population is available	[18]
12	RNA seq based mapping	RNA sequencing based bulked segregant analysis	Mutant and sibling pools	Yes	Applicable to all species where whole genome sequence and mapping population is available	[17]
13	SHOREmap	Mapping by Sequencing	BC_1_F_2_	Yes	Applicable in all where crossing is possible and reference genome is available	[11]
14	deep CAndidateREsequencing (dCARE)	Mapping by Sequencing through BSA	BC_1_F_2_	Yes	Applicable in all where crossing is possible and reference genome is available	[14]
15	Simultaneous Identification of Multiple Causal Mutations (SIMM)	Simultaneous identification of multiple causal mutations in the lines derived from the same parental plant, without requiring a wild- type reference genome. It follows Mapping by sequencing through BSA approach.	BC_1_F_2_	Yes	Identification of causal mutations in multiple mutations at a time by analyzing simultaneously their sequence data. It is Applicable to all.	[24]
16	AgRenSeq	AgRenSeq exploits entire gene set of all strains of a species to isolate the uncharacterized R-genes	Germplasm lines	No	Discovery and cloning of broad range of resistance genes from diverse germplasm	[26]

**Table 4:** List of commonly used bioinformatics software/pipelines for identification and mapping of causal mutations.

S. No.	Name of Pipelines/Softwares/Tools	Data/File Requirements	Used in the Genome of Organism	Web Browser Interface or Standalone Software	Applicability/Usefulness	Source Site/URL	Firstly Designed/Developed by
1	MAQGene	WGS reads in Fastq format	*Caenorhabditis elegans*	Web browser interface	To detect the causative mutations to further classify the mutations based on associated exon annotations	http://maqweb.sourceforge.net	[29]
2	CandiSNP	Whole genome high-throughput sequencing data	*Arabidopsis thaliana*	Web-application	To identify the causal mutations	http://candisnp.tsl.ac.uk	[34]
3	Next-Generation Mapping (NGM)	SNP data from output of either Maq or SAMtools.	*Arabidopsis thaliana*	Web browser interface	To identify causal mutation from F_2_ bulk sequence data.	http://www.bar.utoronto.ca/NGM/index.html	[12]
4	The SNPtrack tool	Paired files having sequencing reads in fastq format	Zebrafish and Mouse	Web browser interface	Mutation mapping in all model systems	http://genetics.bwh.harvard.edu/snptrack	[32]
5	artMAP	Data in BAM or FASTQ formats	*Arabidopsis thaliana*	Standalone software	Identification of EMS-induced mutations in *Arabidopsis*	https://github.com/RihaLab/artMAP	[28]
6	CloudMap	Sequencing data.	*Caenorhabditis elegans* Applicable to other organism also	Web, or cloud or local installation	To detect causal mutations, check for candidate genes, complementation tests.	http://www.usegalaxy.org/cloudmap http://mimodd.readthedocs.io/en/latest/	[33]
7	MASS (Mapping and Assembly with Short Sequences)	Paired end reads obtained from direct sequencing	*Arabidopsis thaliana*	Individual software	Simultaneous mapping and sequencing at a genome-wide level. Identification of a small number of candidate genes/causal mutations within a relatively small interval of 1–2 Mb	http://jcclab.science.oregonstate.edu/MASS	[31]
8	SHORE and SHOREmap	WGS reads in Fastq format, SHOREmap‘interval’ plot and ‘annotate’	*Arabidopsis thaliana*	Web browser interface	To detect the causal mutation site from large pool of recombinant lines	http://1001genomes.org/downloads/shore.html	[11]
9	GenomeMapper	SBS sequencing reads	*Arabidopsis thaliana*	Standalone software	Simultaneous alignments of short reads against multiple genomes	http://1001genomes.org	[30]
10	SIMPLE pipeline	NGS reads in Fastq format	*Arabidopsisthaliana*, *Oryza sativa* L.	Individual software	Implemented for mapping causal mutations in any diploid organism with a sequenced genome	https://github.com/wacguy/Simple	[35]

## References

[1] Chaudhary J., Deshmukh R., Sonah H. (2019). Mutagenesis approaches and their role in crop improvement. Plants.

[2] Cassells A.C., Doyel B.M. (2003). Genetic engineering and mutation breeding for tolerance to abiotic and biotic stresses. Bulg. J. Plant Physiol..

[3] Suprasanna P., Jain S.M. (2017). Mutant Resources and Mutagenomics in crop plants. Emir. J. Food Agric..

[4] Oladosu Y., Rafii Y., Abdullah N., Hussin G., Ramli A., Rahim H.A., Miah G., Usman M. (2016). Principle and application of plant mutagenesis in crop improvement: A review. Biotechnol. Biotechnol. Equip..

[5] Kumawat S., Rana N., Bansal R., Vishwakarma G., Mehetre S.T., Das B.K., Kumar M., Yadav S.K., Sonah H., Sharma T.R. (2019). Expanding avenue of fast neutron mediated mutagenesis for crop improvement. Plants.

[6] Jankowicz-Cieslak J., Mba C., Till B.J., Jankowicz-Cieslak J., Tai T., Kumlehn J., Till B. (2017). Mutagenesis for crop breeding and functional genomics. Biotechnologies for Plant Mutation Breeding.

[7] Pereira R., Oliveira J., Sousa M. (2020). Bioinformatics and computational tools for next-generation sequencing analysis in clinical genetics. J. Clin. Med..

[8] Schneeberger K. (2014). Using next-generation sequencing to isolate mutant genes from forward genetic screens. Nat. Rev. Genet..

[9] Lukowitz W., Gillmor C.S., Scheible W. (2000). Positional Cloning in Arabidopsis. Why It Feels Good to Have a Genome Initiative Working for You. Plant Physiol..

[10] Wilson-Sanchez D., Lup S.D., Sarmiento-Manus R., Ponce M.R., Micol J.L. (2019). Next-generation forward genetic screens: Using simulated data to improve the design of mapping-by-sequencing experiments in Arabidopsis. Nucleic Acids Res..

[11] Schneeberger K., Ossowski S., Lanz C., Juul T., Petersen A.H., Nielsen K.L., Jorgensen J.E., Weigel D., Andersen S.U. (2009). SHOREmap: Simultaneous mapping and mutation identification by deep sequencing. Nat. Methods.

[12] Austin R.S., Vidaurre D., Stamatiou G., Breit R., Provart N.J., Bonetta D. (2011). Next-generation mapping of Arabidopsis genes. Plant J..

[13] Abe A., Kosugi S., Yoshida K., Natsume S., Takagi H., Kanzaki H., Matsumura H., Yoshida K., Mitsuoka C., Tamiru M. (2012). Genome sequencing reveals agronomically important loci in rice using MutMap. Nat. Biotechnol..

[14] Hartwig B., James G.V., Konrad K., Schneeberger K., Turck F. (2012). Fast Isogenic mapping-by-sequencing of ethyl methanesulfonate-induced mutant bulks. Plant Physiol..

[15] Fekih R., Takagi H., Tamiru M., Abe A., Natsume S., Yaegashi H., Sharma S., Sharma S., Kanzaki H., Matsumura H. (2013). MutMap+: Genetic Mapping and Mutant Identification without Crossing in Rice. PLoS ONE.

[16] Takagi H., Uemura A., Yaegashi H., Tamiru M., Abe A., Mitsuoka C., Utsushi H., Natsume S., Kanzaki H., Matsumura H. (2013). MutMap-Gap: Whole-genome resequencing of mutant F_2_ progeny bulk combined with de novo assembly of gap regions identifies the rice blast resistance gene pii. New Phytol..

[17] Miller A.C., Obholzer N.D., Shah A.N., Megason S.G., Moens C.B. (2013). RNA-seq-based mapping and candidate identification of mutations from forward genetic screens. Genome Res..

[18] Takagi H., Abe A., Yoshida K., Kosugi S., Natsume S., Mitsuoka C., Uemura A., Utsushi H., Tamiru M., Takuno S. (2013). QTL-seq: Rapid mapping of quantitative trait loci in rice by whole genome resequencing of DNA from two bulked populations. Plant J..

[19] Mascher M., Richmond T.A., Gerhardt D.J., Himmelbach A., Clissold L., Sampath D., Ayling S., Steuernagel B., Pfeifer M., D’Ascenzo M. (2013). Barley whole exome capture: A tool for genomic research in the genus Hordeum and beyond. Plant J..

[20] Henry I.M., Nagalakshmi U., Lieberman M.C., Ngo K.J., Krasileva K.V., Vasquez-Gross H., Akhunova A., Akhunov E., Dubcovsky J., Tai T.H. (2014). Efficient genome-wide detection and cataloging of EMS-induced mutations using exome capture and next-generation sequencing. Plant Cell.

[21] Nordstrom K., Albani M., James G., Gutjahr C., Hartwig B., Turck F., Paszkowski U., Coupland G., Schneeberger K. (2013). Mutation identification by direct comparison of whole-genome sequencing data from mutant and wild-type individuals using *k*-mers. Nat. Biotechnol..

[22] Sanchez-Martin J., Steuernagel B., Ghosh S., Herren G., Hurni S., Adamski N., Vrana J., Kubalakova M., Krattinger S.G., Wicker T. (2016). Rapid gene isolation in barley and wheat by mutant chromosome sequencing. Genome Biol..

[23] Steuernagel B., Periyannan S., Hernández-Pinzón I., Witek K., Rouse M.N., Yu G., Hatta A., Ayliffe M., Bariana H., Jones J.D.G. (2016). Rapid cloning of disease-resistance genes in plants using mutagenesis and sequence capture. Nat. Biotechnol..

[24] Yan W., Chen Z., Lu J., Xu C., Xie G., Li Y., Deng X.W., He H., Tang X. (2017). Simultaneous Identification of Multiple Causal Mutations in Rice. Front. Plant Sci..

[25] Thind A.K., Wicker T., Simkova H., Fossati D., Moullet O., Brabant C., Vrana J., Doležel J., Krattinger S.G. (2017). Rapid cloning of genes in hexaploid wheat using cultivar-specific long-range chromosome assembly. Nat. Biotechnol..

[26] Arora S., Steuernagel B., Gaurav K., Chandramohan S., Long Y., Matny O. (2019). Resistance gene cloning from a wild crop relative by sequence capture and association genetics. Nat. Biotechnol..

[27] Suchecki R., Sandhu A., Deschamps S., Llaca V., Wolters P., Watson-Haigh N.S., Pallotta M., Whitford R., Baumann U. (2019). LNISKS: Reference-free mutation identification for large and complex crop genomes. bioRxiv.

[28] Javorka P., Raxwal V.K., Najvarek J., Riha K. (2019). artMAP: A user-friendly tool for mapping ethyl methanesulfonate-induced mutations in Arabidopsis. Plant Direct..

[29] Bigelow H., Doitsidou M., Sarin S., Hobert O. (2009). A software tool, MAQGene, facilitating *C. elegans* whole genome sequence analysis for mutant identification. Nat. Methods.

[30] Schneeberger K., Hagmann J., Ossowski J., Warthmann N., Gesing S., Kohlbacher O., Weigel D. (2009). Simultaneous alignment of short reads against multiple genomes. Genome Biol..

[31] Cuperus J.T., Montgomery T.A., Fahlgren N., Burke R.T., Townsend T., Sullivan C.M., Carrington J.C. (2010). Identification of MIR390a precursor processing-defective mutants in Arabidopsis by direct genome sequencing. Proc. Natl. Acad. Sci. USA.

[32] Leshchiner I., Alexa K., Kelsey P., Adzhubei I., Austin-Tse C.A., Cooney J.D., Anderson H., King M.J., Stottmann R.W., Garnaas M.K. (2012). Mutation mapping and identification by whole genome sequencing. Genome Res..

[33] Minevich G., Park D.S., Blankenberg D., Poole R.J., Hobert O. (2012). CloudMap: A cloud-based pipeline for analysis of mutant genome sequences. Genetics.

[34] Etherington G.J., Monaghan J., Zipfel C., Maclean D. (2014). Mapping mutations in plant genomes with the user-friendly web application CandiSNP. Plant Methods.

[35] Wachsman G., Jennifer L.M., Manuel V., Philip N.B. (2017). A SIMPLE Pipeline for Mapping Point Mutations. Plant Physiol..

[36] Kharkwal M.C., Shu Q.Y. (2009). The role of induced mutations in world food security. Induced Plant Mutations in the Genomics Era, Proceedings of the International Joint FAO/IAEA Symposium IAEA, Vienna, Austria, 8–11 June 2009.

[37] Forster B.P., Shu Q.Y., Shu Q.Y., Forster B.P., Nakagawa H. (2011). Plant Mutagenesis in Crop Improvement: Basic Terms and Applications. Plant Mutation Breeding and Biotechnology.

[38] Freisleben R.A., Lein A. (1944). Moglichkeiten und praktischeDurchführung der Mutationszüchtung. Kuhn-Arhiv.

[39] FAO/IAEA, Mutant Variety Database https://mvd.iaea.org/.

[40] Sharma D., Das B.K., Sahu P.K., Tiwari A.K., Baghel S., Sao R., Singh S., Kumar V. (2019). Improvement of Traditional Farmers’ Varieties of Rice through Radiation Induced Mutation Breeding.

[41] Sharma D., Sahu P.K., Das B.K. (2020). BARC-IGKV MoU: A Unique Model of Mutual Collaboration towards Welfare of Farmers’ and Nation.

[42] Sharma D., Das B.K., Kumar V., Tiwari A.K., Sahu P.K., Singh S., Baghel S. (2017). Identification of semi-dwarf and high yielding mutants in Dubraj rice variety of Chhattisgarh through gamma ray based induced mutagenesis. Int. J. Genet..

[43] Martienssen R.A. (1998). Functional genomics: Probing plant gene function and expression with transposons. Proc. Natl. Acad. Sci. USA.

[44] Papworth C., Bauer J.C., Braman J. (1996). QuikChange site-directed mutagenesis. Strategies.

[45] Hemsley A., Arnheim N., Toney M.D., Cortopassi G., Galas D.J. (1989). A simple method for site-directed mutagenesis using the polymerase chain reaction. Nucleic Acids Res..

[46] Serguei P., Mayalagu S., De Y., Yang W.C., Kumaran M., Sundaresan V. (1999). Analysis of Flanking Sequences from Dissociation Insertion Lines: A Database for Reverse Genetics in *Arabidopsis*. Plant Cell..

[47] Takagi H., Tamiru M., Abe A., Yoshida K., Uemura A., Yaegashi H., Obara T., Oikawa K., Utsushi H., Kanzaki E. (2015). MutMap accelerates breeding of a salt-tolerant rice cultivar. Nat. Biotechnol..

[48] Sturtevant A.H. (1913). The Linear Arrangement of Six Sex-Linked Factors in Drosophila, as shown by their mode of Association. J. Exp. Zool..

[49] Mohan M., Nair S., Bhagwat A., Krishna T.G., Yano M., Bhatia C.R., Sasaki T. (1997). Genome mapping, molecular markers and marker-assisted selection in crop plants. Mol. Breed..

[50] Collard B.C.Y., Jahufer M.Z.Z., Brouwer J.B., Pang E.C.K. (2005). An introduction to markers, quantitative trait loci (QTL) mapping and marker-assisted selection for crop improvement: The basic concepts. Euphytica.

[51] Paterson A.H., Lander E.S., Hewitt J.D., Peterson S., Lincoln S.E., Tanksley S.D. (1988). Resolution of quantitative traits into Mendelian factors by using a complete linkage map of restriction fragment length polymorphisms. Nature.

[52] Somers D.J., Isaac P., Edwards K. (2004). A high-density microsatellite consensus map of bread wheat (*Triticum aestivum*). Theor. Appl. Genet..

[53] Milczarski P., Bolibok-Bragoszewska H., Myskow B., Stojalowski S., Heller-Uszynska K., Goralska M., Bragoszewski P., Uszynski G., Kilian A., Rakoczy-Trojanowska M. (2011). A high density consensus map of rye (*Secale cereale* L.) based on DArT markers. PLoS ONE.

[54] Harushima Y., Yano M., Shomura A., Sato M., Shimano T., Kuboki Y., Yamamoto T., Lin S.Y., Antonio B.A., Parco A. (1998). A high-density rice genetic linkage map with 2275 markers using a single F_2_ population. Genetics.

[55] Gupta S.K., Souframanien J., Gopalakrishna T. (2008). Construction of a genetic linkage map in blackgram [*Vigna mungo*(L.) Hepper] based on molecular markers and comparative studies. Genome.

[56] Verma S., Gupta S., Bandhiwal N., Kumar T., Bharadwaj C., Bhatia S. (2015). High-density linkage map construction and mapping of seed trait QTLs in chickpea (*Cicer arietinum* L.) using Genotyping-by-Sequencing (GBS). Sci. Rep..

[57] Muchero W., Ndeye N.D., Bhat P.R., Fenton R.D., Wanamaker S., Pottorff M., Hearne S., Cisse N., Fatokun C., Ehlers J.D. (2009). A consensus genetic map of cowpea [*Vigna unguiculata* (L.) Walp.] and synteny based on EST derived SNPs. Proc. Natl. Acad. Sci. USA.

[58] Song Q., Jenkins J., Jia G., Hyten D.L., Pantalone V., Jackson S.A., Schmutz J., Cregan P.B. (2016). Construction of high resolution genetic linkage maps to improve the soybean genome sequence assembly Glyma1.01. BMC Genom..

[59] Wang X., Yu K., Li H., Peng Q., Chen F., Zhang W., Chen S., Hu M., Zhang J. (2015). High-density SNP map construction and qtl identification for the apetalous character in *Brassica napus* L.. Front. Plant Sci..

[60] Hu X.H., Zhang S.Z., Miao H.R., Cui F.G., Shen Y., Yang W.Q., Xu T.T., Chen N., Chi X.Y., Zhang Z.M. (2018). High-Density Genetic Map Construction and Identification of QTLs Controlling Oleic and Linoleic Acid in Peanut using SLAF-seq and SSRs. Sci. Rep..

[61] Michelmore R.W., Paran I., Kesseli R.V. (1991). Identification of markers linked to disease-resistance genes by bulked segregant analysis—A rapid method to detect markers in specific genomic regions by using segregating populations. Proc. Natl. Acad. Sci. USA.

[62] Zheng Y., Xu F., Li Q., Wang G., Liu N., Gong Y., Li L., Chen Z.H., Xu S. (2018). QTL mapping combined with bulked segregant analysis identify SNP markers linked to leaf shape traits in *Pisum sativum* using SLAF sequencing. Front. Genet..

[63] Govindaraj P., Arumugachamy S., Maheswaran M. (2005). Bulked segregant analysis to detect main effect QTL associated with grain quality parameters in Basmati 370/ASD 16 cross in rice *Oryza sativa* L) using SSR markers. Euphytica.

[64] Gupta S.K., Charpe A., Prabhu K.V., Haque Q.M.R. (2006). Identification and validation of molecular markers linked to the leaf rust resistance gene *Lr19* in wheat. Theor. Appl. Genet..

[65] Muylle H., Baert J., Van Bockstaele E., Moerkerke B., Goetghebeur E., Roldán-Ruiz I. (2005). Identification of molecular markers linked with crown rust (*Puccinia coronata* f. sp. *lolii*) resistance in perennial ryegrass (*Lolium perenne*) using AFLP markers and a bulked segregant approach. Euphytica.

[66] Salunkhe A.S., Poornima R., Prince K.S., Kanagaraj P., Sheeba J.A., Amudha K., Suji K.K., Senthil A., Babu R.C. (2011). Fine mapping QTL for drought resistance traits in rice (*Oryza sativa* L.) using bulk segregant analysis. Mol. Biotechnol..

[67] Huang C., Cui Y., Weng C., Zabel P., Lindhout P. (2000). Development of diagnostic PCR markers closely linked to the tomato powdery mildew resistance gene *Ol-1* on chromosome 6 of tomato. Theor. Appl. Genet..

[68] Wang G.L., Warren R., Innes G., Osborne B., Baker B., Ronald P.C. (1996). Construction of an Arabidopsis BAC library and isolation of clones hybridizing with disease-resistance, gene-like sequences. Plant Mol. Biol. Rep..

[69] Nilmalgoda S.D., Cloutier S., Walichnowski A.Z. (2003). Construction and characterization of a bacterial artificial chromosome (BAC) library of hexaploid wheat (*Triticum aestivum* L.) and validation of genome coverage using locus-specific primers. Genome.

[70] Edwards K.J., Thompson H., Edwards D., de Saizien A., Sparks C., Thompson J.A., Greenland A.J., Eyers M., Schuch W. (1992). Construction and characterisation of a yeast artificial chromosome library containing three haploid maize genome equivalents. Plant Mol. Biol..

[71] Umehara Y., Inagaki A., Tanoue H., Yasukochi Y., Nagamura Y., Saji S., Otsuki Y., Fujimura T., Kurata N., Minobe Y. (1995). Construction and characterization of a rice YAC library for physical mapping. Mol. Breed..

[72] Tomkins J.P., Peterson D.G., Yang T.J., Main D., Wilkins T.A., Paterson A.H., Wing R.A. (2001). Development of genomic resources for cotton (*Gossypium hirsutum* L.): BAC library construction, preliminary STC analysis, and identification of clones associated with fiber development. Mol. Breed..

[73] Park J.Y., Koo D.H., Hong C.P., Lee S.J., Jeon J.W., Lee S.H., Yun P.Y., Park B.S., Kim H.R., Bang J.W. (2005). Physical mapping and microsynteny of *Brassica rapa* ssp. pekinensisgenome corresponding to a 222 kb gene-rich region of Arabidopsis chromosome-4 and partially duplicated on chromosome 5. Mol. Genet. Genom..

[74] Nam Y.W., Penmetsa R.V., Endre G., Uribe P., Kim D., Cool D.R. (1999). Construction of a bacterial artificial chromosome library of *Medicago truncatula* and identification of clones containing ethylene-response gene. Theor. Appl. Genet..

[75] Sanger F., Nicklen S., Coulson A.R. (1977). DNA sequencing with chain terminating inhibitors. Proc. Natl. Acad. Sci. USA.

[76] Maxam A.M., Gilbert W.A. (1977). A new method for sequencing DNA. Proc. Natl. Acad. Sci. USA.

[77] Arabidopsis Genome Initiative (2000). Analysis of the genome sequence of the flowering plant *Arabidopsis thaliana*. Nature.

[78] International Rice Genome Sequencing Project (2005). The map-based sequence of the rice genome. Nature.

[79] Schnable P.S., Ware D., Fulton R.S., Stein J.C., Wei F., Pasternak S., Liang C., Zhang J., Fulton L., Graves T. (2009). The B73 maize genome: Complexity, diversity, and dynamics. Science.

[80] Paterson A.H., Bowers J.E., Bruggmann R., Dubchak I., Grimwood J., Gundlach H., Haberer G., Hellsten U. (2009). The *Sorghum bicolor* genome and the diversification of grasses. Nature.

[81] Schmutz J., Cannon S.B., Schlueter J.A., Ma J., Mitros T., Nelson W., Hyten D.L., Song Q., Thelen J.J., Cheng J. (2010). Genome sequence of the palaeopolyploid soybean. Nature.

[82] Egan A.N., Schlueter J., Spooner D.M. (2012). Applications of next-generation sequencing in plant biology. Am. J. Bot..

[83] Thudi M., Li Y., Jackson S.A., May G.D., Varshney R.K. (2012). Current state-of-art of sequencing technologies for plant genomics research. Brief. Funct. Genom..

[84] Mardis E.R. (2011). A decade’s perspective on DNA sequencing technology. Nature.

[85] Gedil M., Ferguson M., Girma G., Gisel A., Stavolone L., Rabbi I., Kulski J. (2016). Perspectives on the application of next generation sequencing to the improvement of Africa’s staple food crops. Next Generation Sequencing—Advances, Applications and Challenges.

[86] Wang Z., Gerstein M., Snyder M. (2010). RNA-seq: A revolutionary tool for transcriptomics. Nat. Rev. Genet..

[87] Billoud B., Le Bail A., Charrier B. (2008). A stochastic ID nearest-neighbour automaton models early development of the brown alga *Ectocarpus siliculosus*. Funct. Plant Biol..

[88] Sarin S., Prabhu S., O’Meara M.M., Pe’er I., Hobert O. (2008). *Caenorhabditis elegans* mutant allele identification by whole-genome sequencing. Nat. Methods.

[89] Smith D., Quinlan A.R., Peckham H.E., Makowsky K., Tao W., Woolf B., Shen L., Donahue W.F., Tusneem N., Stromberg M.P. (2008). Rapid whole-genome mutational profiling using next-generation sequencing technologies. Genome Res..

[90] Doitsidou M., Jarriault S., Poole R.J. (2016). Next-Generation Sequencing-based Approaches for Mutation Mapping and identification in *Caenorhabditis elegans*. Genetics.

[91] Garg R., Patel R.K., Tyagi A.K., Jain M. (2011). De novo assembly of chickpea transcriptome using short reads for gene discovery and marker identification. DNA Res..

[92] Varshney R.K., Chen W., Li Y., Bharti A.K., Saxena R.K., Schlueter J.A., Donoghue M.T.A., Azam S., Fan G., Whaley A.M. (2012). Draft genome sequence of pigeonpea (*Cajanus cajan*), an orphan legume crop of resource-poor farmers. Nat. Biotechnol..

[93] Henry R.J. (2012). Next-generation sequencing for understanding and accelerating crop domestication. Brief. Funct. Genom..

[94] Elshire R.J., Glaubitz J.C., Sun Q., Poland J.A., Kawamoto K., Buckler E.S., Mitchell S.E. (2011). A robust, simple genotyping-by-sequencing (GBS) approach for high diversity species. PLoS ONE.

[95] Tabata R., Kamiya T., Shigenobu S., Yamaguchi K., Yamada M., Hasebe M., Fujiwara T., Sawa S. (2013). Identification of an EMS-induced causal mutation in a gene required for boron-mediated root development by low-coverage genome re-sequencing in *Arabidopsis*. Plant Signal Behav..

[96] Salvi S., Tuberosa R. (2005). To clone or not to clone plant QTLs: Present and future challenges. Trends Plant Sci..

[97] Huang X., Feng Q., Qian Q., Zhao Q., Wang L., Wang A., Guan J., Fan D., Weng Q., Huang T. (2009). High-throughput genotyping by whole genome resequencing. Genome Res..

[98] Spindel J., Wright M., Chen C., Cobb J., Gage J., Harrington S., Lorieux M., Ahmadi N., McCouch S. (2013). Bridging the genotyping gap: Using genotyping by sequencing (GBS) to add high-density SNP markers and new value to traditional bi-parental mapping and breeding populations. Theor. Appl. Genet..

[99] Chapman J.A., Mascher M., Buluc A., Barry K., Georganas E., Session A., Strnadova V., Jenkins J., Sehgal S., Oliker L. (2015). A whole-genome shotgun approach for assembling and anchoring the hexaploid bread wheat genome. Genome Biol..

[100] Nguyen K.L., Grondin A., Courtois B., Gantet P. (2019). Next-Generation Sequencing Accelerates Crop Gene Discovery. Trends Plant Sci..

[101] Lander E.S., Linton L.M., Birren B., Nusbaum C., Zody M.C., Baldwin J., Devon K., Dewar K., Doyle M., FitzHugh W. (2001). Initial sequencing and analysis of the human genome. Nature.

[102] Gelli M., Mitchell S.E., Liu K., Clemente T.E., Weeks D.P., Zhang C., Holding D.R., Dweikat I.M. (2016). Mapping QTLs and association of differentially expressed gene transcripts for multiple agronomic traits under different nitrogen levels in sorghum. BMC Plant Biol..

[103] Jiang Q., Tang D., Hu C., Qu J., Liu J. (2016). Combining meta-QTL with RNA-seq data toidentify candidate genes ofkernel row number trait in maize. Maydica.

[104] Qi X., Li M., Xie M., Liu X., Ni M., Shao G., Song C., Yim A.K., Tao Y., Wong F. (2014). Identification of a novel salt tolerance gene in wildsoybean by whole-genome sequencing. Nat. Commun..

[105] Majewski J., Pastinen T. (2011). The study of eQTL variations by RNA-seq: From SNPs to phenotypes. Trends Genet..

[106] Westra H., Franke L. (2014). From genome to function by studying eQTLs. Biochim. Biophys. Acta.

[107] Sun H., Schneeberger K. (2015). SHOREmap v3.0: Fast and accurate identification of causal mutations from forward genetic screens. Methods Mol. Biol..

[108] Wang H., Zhang Y., Sun L., Xu P., Tu R., Meng S., Wu W., Anis G.B., Hussain K., Riaz A. (2018). *WB1*, a Regulator of Endosperm Development in Rice, Is Identified by a Modified MutMap Method. Int. J. Mol. Sci..

[109] Yuan Y., Bayer P.E., Batley J., Edwards D. (2017). Improvements in genomic technologies: Application to crop genomics. Trends Biotechnol..

[110] Han X., Xu R., Duan P., Yu H., Luo Y., Li Y. (2017). Genetic analysis and identification of candidate genes for two spotted-leaf mutants (*spl101* and *spl102*) in rice. Hereditas.

[111] Deng L., Qin P., Liu Z., Wang G., Chen W., Tong J., Xiao L., Tu B., Sun Y., Yan W. (2017). Characterization and fine-mapping of a novel premature leaf senescence mutant *yellow leaf and dwarf 1* in rice. Plant Physiol. Biochem..

[112] Hu Y.G., Guo L., Yang G.T., Qin P., Fan C.L., Peng Y.L., Yan W., He H., Li S.G. (2016). Genetic analysis of dense and erect panicle-2 allele DEP2-1388 and its application in hybrid rice breeding. Hereditas.

[113] Cao Z.Z., Lin X.Y., Yang Y.J., Guan M.Y., Xu P., Chen M.X. (2019). Gene identification and transcriptome analysis of low cadmium accumulation rice mutant (*lcd1*) in response to cadmium stress using MutMap and RNA-seq. BMC Plant Biol..

[114] Zhang H., Li M., He D., Wang K., Yang P. (2020). Mutations on *ent*-kaurene oxidase 1 encoding gene attenuate its enzyme activity of catalyzing the reaction from *ent*-kaurene to *ent*-kaurenoic acid and lead to delayed germination in rice. PLoS Genet..

[115] Chen J., Huang X.Y., Salt D.E., Zhao F.J. (2020). Mutation in OsCADT1 enhances cadmium tolerance and enriches selenium in rice grain. New Phytol..

[116] Sathe A.P., Su X., Chen Z., Chen T., Wei X., Tang S., Zhang X., Wu J. (2019). Identification and characterization of a spotted-leaf mutant spl40 with enhanced bacterial blight resistance in rice. Rice.

[117] Zhang Y., Liu Q., Zhang Y., Chen Y., Yu N., Cao Y., Zhan X., Cheng S., Cao L. (2019). *LMM24* Encodes Receptor-Like Cytoplasmic Kinase 109, Which Regulates Cell Death and Defense Responses in Rice. Int. J. Mol. Sci..

[118] Jiang L., Ma X., Zhao S., Tang Y., Liu F., Gu P., Fu Y., Zhu Z., Cai H., Sun C. (2019). The APETALA2-Like Transcription Factor SUPERNUMERARY BRACT Controls Rice Seed Shattering and Seed Size. Plant Cell.

[119] Wang Z., Li J., Chen S., Heng Y., Chen Z., Yang J., Zhou K., Pei J., He H., Deng X.W. (2017). Poaceae-specific *MS1* encodes a phospholipid-binding protein for male fertility in bread wheat. Proc. Natl. Acad. Sci. USA.

[120] Chen J., Jiang Y., Laza H., Payton P., Ware D., Xin Z. (2019). Identification of the First Nuclear Male Sterility Gene (Male-sterile 9) in Sorghum. Plant Genome.

[121] Jiao Y., Burow G., Gladman N., Martinez V.A., Chen J., Burke J., Ware D., Xin Z. (2018). Efficient identification of causal mutations through sequencing of bulked F_2_ from two allelic bloomless mutants of *Sorghum bicolor*. Front. Plant Sci..

[122] Cheng H., Jin F., Zaman Q.U., Ding B., Hao M., Wang Y., Huang Y., Wells R., Dong Y., Hu Q. (2019). Identification of Bna.IAA7.C05 as allelic gene for dwarf mutant generated from tissue culture in oilseed rape. BMC Plant Biol..

[123] Tran Q.H., Bui N.H., Kappel C., Dau N.T.N., Nguyen L.T., Tran T.T., Khanh T.D., Trung K.H., Lenhard M., Vi S.L. (2020). Mapping-by-Sequencing via MutMap Identifies a Mutation in *ZmCLE7* Underlying Fasciation in a Newly Developed EMS Mutant Population in an Elite Tropical Maize Inbred. Genes.

[124] Klein H., Xiao Y., Conklin P.A., Govindarajulu R., Kelly J.A., Scanlon M.J., Whipple C.J., Bartlett M. (2018). Bulked-Segregant Analysis Coupled to Whole Genome Sequencing (BSA-Seq) for Rapid Gene Cloning in Maize. G3.

[125] Amin G.M., Kong K., Sharmin R.A., Kong J., Bhat J.A., Zhao T. (2019). Characterization and Rapid Gene-Mapping of Leaf Lesion Mimic Phenotype of *spl-1* Mutant in Soybean (*Glycine max* (L.) Merr.). Int. J. Mol. Sci..

[126] Takagi H., Abe A., Uemura A., Oikawa K., Utsushi H., Yaegashi H., Kikuchi H., Shimizu M., Abe Y., Kanzaki H. (2017). Rice blast resistance gene *Pii* is controlled by a pair of NBS-LRR genes *Pii-1* and *Pii-2*. bioRxiv.

[127] Nakata M., Miyashita T., Kimura R., Nakata Y., Takagi H., Kuroda M., Yamaguchi T., Umemoto T., Yamakawa H. (2018). MutMapPlus identified novel mutant alleles of a rice starch branching enzyme II b gene for fine-tuning of cooked rice texture. Plant Biotechnol..

[128] Imamura T., Takagi H., Miyazato A., Ohki S., Mizukoshi H., Mori M. (2018). Isolation and characterization of the betalain biosynthesis gene involved in hypocotyl pigmentation of the allotetraploid *Chenopodium quinoa*. Biochem. Biophys. Res. Commun..

[129] Zou T., Xiao Q., Li W., Luo T., Yuan G., He Z., Liu M., Li Q., Xu P., Zhu J. (2017). OsLAP6/OsPKS1, an orthologue of *Arabidopsis* PKSA/LAP6, is critical for proper pollen exine formation. Rice.

[130] Dracatos P.M., Bartos J., Elmansour H., Singh D., Karafiatova M., Zhang P., Steuernagel B., Svacina R., Cobbin J.C.A., Clark B. (2019). The Coiled-Coil NLR *Rph1*, confers leaf rust resistance in barley cultivar sudan. Plant Physiol..

[131] Marchal C., Zhang J., Zhang P., Fenwick P., Steuernagel B., Adamski N.M., Boyd L., McIntosh R., Wulff B., Berry S. (2018). BED-domain-containing immune receptors confer diverse resistance spectra to yellow rust. Nat. Plants.

[132] Mo Y., Howell T., Vasquez-Gross H., de Haro L.A., Dubcovsky J., Pearce S. (2018). Mapping causal mutations by exome sequencing in a wheat TILLING population: A tall mutant case study. Mol. Genet. Genom..

[133] Hussain M., Iqbal M.A., Till B.J., Rahman M. (2018). Identification of induced mutations in hexaploid wheat genome using exome capture assay. PLoS ONE.

[134] Saintenac C., Jiang D., Akhunov E.D. (2011). Targeted analysis of nucleotide and copy number variation by exon capture in allotetraploid wheat genome. Genome Biol..

[135] Das S., Upadhyaya H.D., Bajaj D., Kujur A., Badoni S., Kumar V., Tripathi S., Gowda C.L.L., Sharma S., Singh S. (2015). Deploying QTL-seq for rapid delineation of a potential candidate gene underlying major trait-associated QTL in chickpea. DNA Res..

[136] Lu H., Lin T., Klein J., Wang S., Qi J., Zhou Q., Sun J., Zhang Z., Weng Y., Huang S. (2014). QTL-seq identifies an early flowering QTL located near flowering locus T. in cucumber. Theor. Appl. Genet..

[137] Yaobin Q., Peng C., Yichen C., Yue F., Derun H., Tingxu H., Xianjun S., Jiezheng Y. (2018). QTL-Seq identified a major QTL for grain length and weight in rice using near isogenic F_2_ population. Rice Sci..

[138] Zhang X., Wang W., Guo N., Zhang Y., Bu Y., Zhao J., Xing H. (2018). Combining QTL-seq and linkage mapping to fine map a wild soybean allele characteristic of greater plant height. BMC Genom..

[139] Ramos A., Fu Y., Michael V., Meru G. (2020). QTL-seq for identification of loci associated with resistance to Phytophthora crown rot in squash. Sci. Rep..

[140] Edae E.A., Rouse M.N. (2019). Bulked segregant analysis RNA-seq (BSR-Seq) validated a stem resistance locus in *Aegilops umbellulata*, a wild relative of wheat. PLoS ONE.

[141] Nishijima R., Yoshida K., Sakaguchi K., Yoshimura S.I., Sato K., Takumi S. (2018). RNA sequencing-based bulked segregant analysis facilitates efficient D-genome marker development for a specific chromosomal region of synthetic hexaploid wheat. Int. J. Mol. Sci..

[142] Thakur V., Wanchana S., Banerjee R., Kumar G.V., Kumar S.P.J. (2019). Gene Discovery by Forward Genetic Approach in the Era of High-Throughput Sequencing. OMICS-Based Approaches in Plant Biotechnology. OMICS-BasedApproaches in Plant Biotechnology.

[143] Li H., Ruan J., Durbin R. (2008). Mapping short DNA sequencing reads and calling variants using mapping quality scores. Genome Res..

[144] Singh V.K., Khan A.W., Saxena R.K., Kumar V., Kale S.M., Sinha P., Chitikineni A., Pazhamala L.T., Garg V., Sharma M. (2016). Next-generation sequencing for identification of candidate genes for Fusarium wilt and sterility mosaic disease in pigeonpea (*Cajanus cajan*). Plant Biotechnol. J..

[145] Weber A.P.M. (2015). Discovering new biology through sequencing of RNA. Plant Physiol..

[146] Giovannoni J.J., Wing R.A., Ganal M.W., Tanksley S.D. (1991). Isolation of molecular markers from specific chromosomal intervals using DNA pools from existing mapping populations. Nucleic Acids Res..

[147] Illa-Berenguer E., Van Houten J., Huang Z., Van der Knaap E. (2015). Rapid and reliable identification of tomato fruit weight and locule number loci by QTL-seq. Theor. Appl. Genet..

[148] Singh V.K., Khan A.W., Jaganathan D., Thudi M., Roorkiwal M., Takagi H., Garg V., Kumar V., Chitikineni A., Gaur P.M. (2016). QTL-seq for rapid identification of candidate genes for 100-seed weight and root/total plant dry weight ratio under rainfed conditions in chickpea. Plant Biotechnol. J..

[149] Clevenger J., Chu Y., Chavarro C., Botton S., Culbreath A., Isleib T.G., Holbrook C.C., Ozias-Akins P. (2018). Mapping late leaf spot resistance in peanut (*Arachis hypogaea*) using QTL-seq reveals markers for marker-assisted selection. Front. Plant Sci..

[150] Branham S.E. (2018). QTL-seq and marker development for resistance to *Fusarium oxysporum* f. sp. *niveum* race 1 in cultivated watermelon. Mol. Breed..

[151] Fall L.A., Clevenger J., McGregor C. (2018). Assay development and marker validation for marker assisted selection of *Fusarium oxysporum* f. sp. *niveum* race 1 in watermelon. Mol. Breed..

[152] Shu J., Liu Y., Zhang L., Li Z., Fang Z., Yang L., Zhuang M., Zhang Y., Lv H. (2018). QTL-seq for rapid identification of candidate genes for flowering time in broccoli × cabbage. Theor. Appl. Genet..

[153] Branham S.E., Farnham M.W. (2019). Identification of heat tolerance loci in broccoli through bulked segregant analysis using whole genome resequencing. Euphytica.

[154] Warr A., Robert C., Hume D., Archibald A., Deeb N., Watson M. (2015). Exome sequencing: Current and future perspectives. G3 Genes Genomes Genet..

[155] Krasileva K.V., Vasquez-Gross H.A., Howell T., Bailey P., Paraiso F., Clissold L., Simmonds J., Ramirez-Gonzalez R.H., Wang X., Borrill P. (2017). Uncovering hidden variation in polyploid wheat. Proc. Natl. Acad. Sci. USA.

[156] Allen R.S., Nakasugi K., Doran R.L., Millar A.A., Waterhouse P.M. (2013). Facile mutant identification via a single parental backcross method and application of whole genome sequencing based mapping pipelines. Front. Plant Sci..

[157] Kaur P., Gaikwad K. (2017). From Genomes to GENE-omes: Exome Sequencing Concept and Applications in Crop Improvement. Front. Plant Sci..

[158] Marcais G., Kingsford C.A. (2011). Fast, lock-free approach for efficient parallel counting of occurrences of k-mers. Bioinformatics.

[159] Feuillet C., Travella S., Stein N., Albar L., Nublat A., Keller B. (2003). Map-based isolation of the leaf rust disease resistance gene *Lr10* from the hexaploid wheat (*Triticum aestivum* L.) genome. Proc. Natl. Acad. Sci. USA.

[160] Rabinowicz P.D. (2003). Constructing gene enriched plant genomic libraries using methylation filtration technology. Methods Mol. Biol..

[161] Shagina I., Bogdanova E., Mamedov I.Z., Lebedev Y., Lukyanov S., Shagin D. (2010). Normalization of genomic DNA using duplex-specific nuclease. BioTechniques.

[162] Oono Y., Kobayashi F., Kawahara Y., Yazawa T., Handa H., Itoh T., Matsumoto T. (2013). Characterization of the wheat (*Triticum aestivum* L.) transcriptome by de novo assembly for the discovery of phosphate starvation-responsive genes: Gene expression in pi-stressed wheat. BMC Genom..

[163] Steuernagel B., Vrana J., Karafiatova M., Wulff B.B.H., Dolezel J., Periyannan S. (2017). Rapid gene isolation using MutChromSeq. Wheat Rust Diseases: Methods in Molecular Biology.

[164] Jupe F., Chen X., Verweij W., Witek K., Jones J.D., Hein I. (2014). Genomic DNA library preparation for resistance gene enrichment and sequencing (RenSeq) in plants. Methods Mol. Biol..

[165] Li R., Yu C., Li Y., Lam T.W., Yiu S.M., Kristiansen K., Wang J. (2009). SOAP2: An improved ultrafast tool for short read alignment. Bioinformatics.

[166] Li H., Durbin R. (2009). Fast and accurate short read alignment with Burrows-Wheeler transform. Bioinformatics.

[167] Langmead B., Salzberg S.L. (2012). Fast gapped-read alignment with Bowtie-2. Nat. Methods..

[168] Li R., Li Y., Fang X., Yang H., Wang J., Kristiansen K., Wang J. (2009). SNP detection for massively parallel whole-genome resequencing. Genome Res..

[169] Hill J.T., Demarest B.L., Bisgrove B.W., Gorsi B., Su Y.C., Yost H.J. (2013). MMAPPR: Mutation mapping analysis pipeline for pooled RNA-seq. Genome Res..

[170] Lochlainn S.O., Amoah S., Graham N.S., Alamer K., Rios J.J., Kurup S., Stoute A., Hammond J.P., Ostergaard L., King G.J. (2011). High resolution melt (HRM) analysis is an efficient tool to genotype EMS mutant sincomplex crop genomes. Plant Methods.

[171] Pan X., Yan W., Chang Z., Xu Y., Luo M., Xu C., Chen Z. (2020). *OsMYB80* regulates anther development and pollen fertility by targeting multiple biological pathways. Plant Cell Physiol..

[172] Lyu J. (2019). A new mapping strategy. Nat. Plants.

[173] Jaganathan D., Bohra A., Thudi M., Varshney R.K. (2020). Fine mapping and gene cloning in the post-NGS era: Advances and prospects. Theor. Appl. Genet..

[174] Candela H., Casanova-Sez R., Micol J.L. (2015). Getting started in mapping-by-sequencing. J. Integr. Plant Biol..

[175] Jordan K.W., Wang S., Lun Y., Gardiner L.J., MacLachlan R., Hucl P., Wiebe K., Wong D., Forrest K.L., Sharpe A.G. (2015). A haplotype map of allohexaploid wheat reveals distinct patterns of selection on homoeologous genomes. Genome Biol..

[176] Zhang J., Chiodini R., Badr A., Zhang G. (2011). The impact of next-generation sequencing on genomics. J. Genet. Genom..

[177] Weigel D., Mott R. (2009). The 1001 Genomes Project for Arabidopsis thaliana. Genome Biol..

[178] Ossowski S., Schneeberger K., Clark R.M., Lanz C., Warthmann N., Weigel D. (2008). Sequencing of natural strains of *Arabidopsis thaliana* with short reads. Genome Res..

[179] Dong Z., Han M.H., Fedoroff N. (2008). The RNA-binding proteins HYL1 and SE promote accurate in vitro processing of pri-miRNA by DCL1. Proc. Natl. Acad. Sci. USA.

[180] Hiraguri A., Itoh R., Kondo N., Nomura Y., Aizawa D., Murai Y., Koiwa H., Seki M.H., Shinozaki K., Fukuhara T. (2005). Specific interactions between Dicer-like proteins and HYL1/DRB family dsRNA-binding proteins in *Arabidopsis thaliana*. Plant Mol. Biol..

[181] Schmitt M.W., Kennedy S.R., Salk J.J., Fox E.J., Hiatt J.B., Loeb L.A. (2012). Detection of ultra-rare mutations by next-generation sequencing. Proc. Natl. Acad. Sci. USA.

[182] Xu J., Kelly R., Zhou G., Turner S.A., Ding D., Harris S.C., Hong H., Fang H., Tong W. (2012). SNPTrack: An integrated bioinformatics system for genetic association studies. Hum. Genom..

[183] Marjoram L., Alvers A., Deerhake M.E., Bagwell J., Mankiewicz J., Cocchiaro J.L., Beerman R.W., Willer J., Sumigray K.D., Katsanis N. (2015). Epigenetic control of intestinal barrier function and inflammation in zebrafish. Proc. Natl. Acad. Sci. USA.

[184] Liberman L.M., Sparks E.E., Moreno-Risueno M.A., Petricka J.J., Benfey P.N. (2015). MYB36 regulates the transition from proliferation to differentiation in the *Arabidopsis* root. Proc. Natl. Acad. Sci. USA.

[185] Cingolani P., Platts A., le Wang L., Coon M., Nguyen T., Wang L., Land S.J., Lu X., Ruden D.M. (2012). A program for annotating and predicting the effects of single nucleotide polymorphisms, SnpEff: SNPs in the genome of *Drosophila melanogaster* strain w1118; iso-2; iso-3. Fly.

[186] Wambugu P., Ndjiondjop M.N., Furtado A., Henry R. (2018). Sequencing of bulks of segregants allows dissection of genetic control of amylose content in rice. Plant Biotechnol. J..

[187] Xu D., Dhiman R., Garibay A., Mock H.P., Leister D., Kleine T. (2020). Cellulose defects in the *Arabidopsis* secondary cell wall promote early chloroplast development. Plant J..

[188] Voelkerding K.V., Dames S., Durtschi J.D. (2010). Next generation sequencing for clinical diagnostics—Principles and application to targeted resequencing for hypertrophic cardiomyopathy. J. Mol. Diagn..

[189] Alkan C., Sajjadian S., Eichler E.E. (2011). Limitations of next-generation genome sequence assembly. Nat. Methods.

[190] Jo Y.D., Kim J.B. (2019). Frequency and Spectrum of Radiation-Induced Mutations Revealed by Whole-Genome Sequencing Analyses of Plants. Quantum Beam Sci..

[191] Stahlberg A., Paul M., Krzyzanowski P.M., Jackson J.B., Egyud M., Stein L., Godfrey T.E. (2016). Simple, multiplexed, PCR-based barcoding of DNA enables sensitive mutation detection, in liquid biopsies using sequencing. Nucleic Acids Res..

[192] Stahlberg A., Krzyzanowski P.M., Egyud M., Filges S., Stein L., Godfrey T.E. (2017). Simple multiplexed PCR-based barcoding of DNA for ultrasensitive mutation detection by next-generation sequencing. Nat. Protoc..

[193] Monson-Miller J., Sanchez-Mendez D.C., Fass J., Henry I.M., Tai T.H., Comai L. (2012). Reference genome-independent assessment of mutation density using restriction enzyme-phased sequencing. BMC Genom..

[194] Vlk D., Repkova J. (2017). Application of Next-Generation Sequencing in Plant Breeding. Czech J. Genet. Plant Breed..

